# Accurate Prediction of Protein Catalytic Residues by Side Chain Orientation and Residue Contact Density

**DOI:** 10.1371/journal.pone.0047951

**Published:** 2012-10-24

**Authors:** Yu-Tung Chien, Shao-Wei Huang

**Affiliations:** Department of Medical Informatics, Tzu Chi University, Hualien, Taiwan, Republic of China; Indian Institute of Science, India

## Abstract

Prediction of protein catalytic residues provides useful information for the studies of protein functions. Most of the existing methods combine both structure and sequence information but heavily rely on sequence conservation from multiple sequence alignments. The contribution of structure information is usually less than that of sequence conservation in existing methods. We found a novel structure feature, residue side chain orientation, which is the first structure-based feature that achieves prediction results comparable to that of evolutionary sequence conservation. We developed a structure-based method, Enzyme Catalytic residue SIde-chain Arrangement (EXIA), which is based on residue side chain orientations and backbone flexibility of protein structure. The prediction that uses EXIA outperforms existing structure-based features. The prediction quality of combing EXIA and sequence conservation exceeds that of the state-of-the-art prediction methods. EXIA is designed to predict catalytic residues from *single* protein structure without needing sequence or structure alignments. It provides invaluable information when there is no sufficient or reliable homology information for target protein. We found that catalytic residues have very special side chain orientation and designed the EXIA method based on the newly discovered feature. It was also found that EXIA performs well for a dataset of enzymes without any bounded ligand in their crystallographic structures.

## Introduction

Due to the advances of structural genomics project, the number of protein structures determined is increasing rapidly. However, the functions and catalytic mechanisms of a huge number of proteins remain unclear because of the time-consuming processes of wet-lab experimental approaches. It becomes increasingly important to predict catalytic residues by *computation* methods, which can greatly reduce the time and costs for researchers. Prediction of catalytic residues is challenging because of the fact that only a small fraction of residues in protein are catalytic residues. Despite the number of catalytic residues is small in proteins, they are directly involved in catalytic reactions and play an important role in enzyme catalysis.

Many methods have been proposed to predict protein catalytic residues from its sequence or structure. The most direct strategy is to find its homologous sequences or structures whose function and catalytic residues are already known [1]–[5]. An information-theoretic approach for estimating sequence conservation based on Jensen–Shannon divergence was used to predict catalytic residues from protein sequence [1]. Phylogenetic motifs, sequence regions conserving the overall familial phylogeny was shown to be a promising feature for protein functional site prediction [2]. Sequence conservation and 3D-profile, including cleft shape, stability, and electrostatic potential, generated from known enzyme structures was used to identify catalytic sites [3]. Another method detects specific conservation patterns near known catalytic residues on sequence and constrains what combination of amino acids can exist near a predicted catalytic residue [4]. A library of structural templates representing catalytic sites, based on information from literatures, and analysis of homologous template families were used to locate catalytic sites [5]. Neural network combined with sequence identity and sequence conservation was demonstrated to be able to accurately predict enzyme catalytic residues [6]. Another work used not only sequence conservation, but also predicted secondary structures and predicted solvent accessible surface [7]. Catalytic residues are identified by multiple sequence alignment or structure template search with enzymes whose catalytic residues are already annotated. However, there are limitations for such homology-based methods. First, homologous enzymes whose catalytic residues are already correctly annotated are required. Second, proteins that have similar structure do not always have identical catalytic residues [8]. There are also situations that proteins of the same function have quite different tertiary structures [9].

Another group of methods directly identify catalytic sites from protein structure without relying on sequence conservation from multiple sequence alignments or structure template search. They aim to find out the fundamental different characteristics between catalytic residues and noncatalytic residues. It was found that, if a protein was represented as a network in which the residues are vertices and their interactions are edges, the central residues, i.e. the central hubs are usually functional important residues or in direct contact with them [10]. It was also reported that the catalytic residues are usually located in small fractions of the exposed residues closest to the protein centroid [11]. The calculation of a force constant, i.e. the ease of moving a given residue with respect to the other residues in the protein, was applied to the detection of catalytic residues. It was concluded that catalytic residues usually have higher force constant [12]. A method called Theoretical Microscopic Titration Curves (THEMATICS) [13] was developed to predict catalytic residues by computing residue electrostatic properties from protein structure. THEMATICS was later combined with geometric features derived from protein structure [14] to predict catalytic residues from enzyme structure using a monotonicity-constrained maximum likelihood approach, called Partial Order Optimum Likelihood (POOL). A more recent study [15] models spherical regions around target residues, extracts the properties of their content such as physico-chemical properties, atomic density, flexibility, presence of water molecules or heteroatoms. These extracted features are combined with sequence information, including sequence conservation.

We propose a structure-based method (EXIA) that predicts catalytic residues from single protein structure without needing sequence or structure alignments. The novelty of EXIA is based on calculating orientation of side chain vectors, which is a newly found unique structural feature of catalytic residues. The proposed method is compared against existing structure-based features and has the best performance among these structure-based features. EXIA method combined with sequence conservation from PSI-Blast outperforms state-of-the-art catalytic residue prediction methods. In addition to prediction, the finding also benefits to understanding of the special structural features of catalytic residues.

## Methods

### Overview of the Prediction System

**Figure 1:**
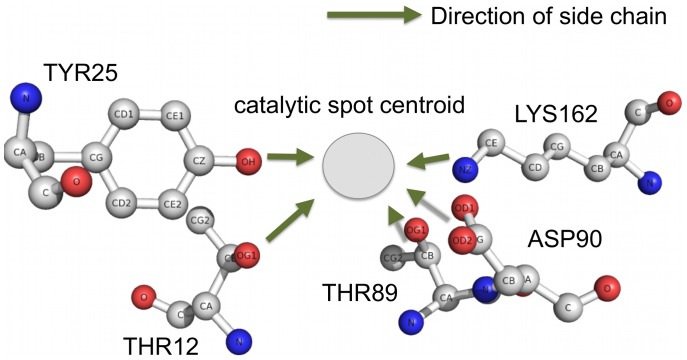
Structures of the catalytic residues of *E-coli* asparaginase II. Structures of the catalytic residues of *E-coli* asparaginase II (PDB id: 3eca). Thr12, Tyr25, Thr89, Asp90, and Lys162, in chain A of this protein form a catalytic spot (ligands and other residues not shown). Residue side chain direction is defined as the vector from its Cα atom to its functional atom. The side chain vectors of catalytic residues tend to point to the center region of the catalytic spot. “Catalytic spot centroid” is simply a concept and is not formally defined in EXIA method. This observation and the concept are the basic ideas of the EXIA method.

**Figure 2:**
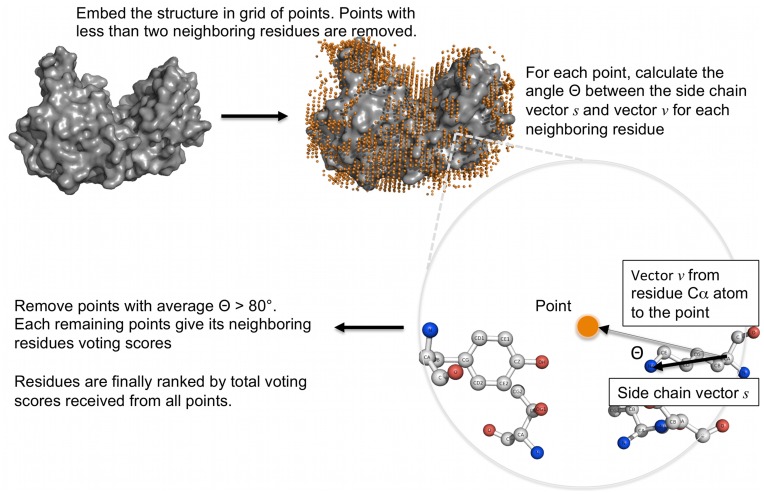
Overview of the EXIA method. The protein structure is first embedded in grid of points. Points with less than two neighboring residues (distance <10 Å) are removed. Then for each neighboring residue of each remaining point, the angle between the side chain vector of the residue and the vector from residues Cα atom to the position of the point is calculated. The points with average angle (average angle of all its neighboring residues) <80° are removed. Each remaining point gives its neighboring residues a voting score based on backbone flexibility. In the end, residues are ranked by their total voted scores.

The idea of the method comes from the fact that most enzyme catalysis is the collaboration between multiple catalytic residues that form a “catalytic spot”. The catalytic residue functional part, i.e. the atoms directly participating in catalytic reaction, is usually located on the side chain. We found that the vector between the Cα atom and the functional atom, i.e. the direction of side chain, usually points to the center of the catalytic spot. Figure 1 illustrates the phenomenon in *E-coli* asparaginase II (PDB id: 3eca). The catalytic residues, T12, Y25, T89, D90, and K162, in chain A of this protein form a catalytic spot (ligands and other residues not shown). The side chains all point to the center of the catalytic spot. The EXIA method is designed based on this observation and is summarized in Figure 2.

There are two phases in the method: in the first phase, only residue types whose functional part locating on the side chain are included in the calculation; in the second phase, residues of other types are predicted based on the results of the first phase.

### Definition of Side Chain Vector

The side chain direction of residue *k* is the vector 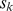 from its Cα atom to its functional atom:
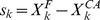
(1)where 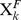 and 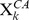 are the crystallographic position of the functional atom and Cα atom of residue *k*. The definitions are based on the annotations in Catalytic Site Atlas (CSA) [16]. The most frequent functional atom for each amino acid is used. If there were more than one frequent functional atom, the atom closest to the centroid of functional atoms is used. For example, arginine has two frequent functional atoms, NH1 and NH2. Instead of using NH1 or NH2, we used atom CZ that is closest to the centroid of NH1 and NH2. Table 1 lists the amino acid types whose functional atom is on the side chain (side chain functional amino acids) and the atom we decided to use to calculate their side chain vector.

**Table 1:** List of side chain vector atoms.

Amino acid type	Side chain vector atom^1^
ARG	CZ
ASN	CG
ASP	CG
CYS	SG
GLN	CD
GLU	CD
HIS	NE
LYS	NZ
SER	OG
THR	OG
TRP	CZ
TYR	OH

1 The side chain vector is from residue Cα atom to its side chain vector atom. Atom nomenclatures are from Protein data bank [28].

### First Phase – Predicting Side Chain Functional Amino Acids

In the first phase, only amino acids listed in Table 1 are included in calculations of side chain directions. Other residues are not removed from the structure but are only used in the calculation of backbone flexibility. First, the structure is embedded in a three-dimensional 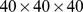 grid of points. Each point is a probable position of the catalytic spot. The grid size is the optimal balance between program speed and grid spacing small enough to scan possible spots. The grid spacing is between 1 Å to 1.6 Å depending on the protein size. The prediction performance is worse using larger grid spacing. Using smaller grid spacing spent more computation time but would not improve prediction performance. For each point *i*, residues having a distance between its Cα atom and the point *i* less than 10Å are defined as the surrounding residues of point *i*. Points that have less than three surrounding residues are removed. For each point *i* and any one of its surrounding residue *j*, the vector between point *i* and Cα atom of residue *j* is defined as:
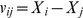
(2)where 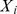 and 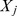 are the position of point *i* and Cα atom of residue *j*. We compute the angle 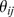 between 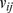 and 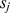, which is the side chain vector of residue *j*,



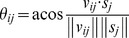
(3)For points that are within or near the area of the catalytic spot, they should have smaller 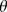 angles. For each point *i*, the averaged angle 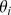 among all of its surrounding residues is defined as,
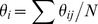
(4)where *N* is the number of surrounding residues of point *i*. We assume that points near the catalytic spot have smaller averaged 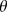 and the points that have averaged 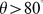 are removed. The cut-off value is chosen based on the statistics of side chain orientations of catalytic residues (as shown in the “Analysis on side chain orientations of catalytic residues” section). About 80% of catalytic residues have the angle θ ≤80 degrees. We found that it is the most proper cut-off value by trying different cut-off values ranging from 30 to 100 degrees. For every remaining point with *N* surrounding residues, we select three residues from *N* surrounding residues and give each selected residue a “voting score”. For each point, the selection process is repeated for all possible combinations of any three surrounding residues. Residues are finally ranked by their sum of voting scores (denoted as 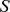) received from these points. The final result of this phase is a list of residues ranked by their 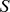 score, i.e. the likelihood of being a catalytic residue according to our prediction. The design of voting score is described in the next section.

### Voting Score

The voting score is based on the weighted-contact number model (WCN) [17], [18], which is a measure of backbone flexibility of residues. The WCN model was applied to the study of structural characteristics of catalytic residues. Catalytic residues usually have high WCN, i.e. structurally more rigid [19]. For any residue *k* in a structure, its WCN 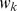 is defined as,
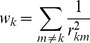
(5)where *m* is any other residues in the structure and 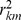 is the squared distance between the Cα atoms of residue *k* and *m*. This calculation includes the Cα atoms of all residues in the structure (not limited to residues of amino acid types listed in Table 1). As described in the previous section, for every remaining point with *N* surrounding residues, we select three residues from its *N* surrounding residues and give each selected residue a voting score. The voting strategy not only gives higher score to residues involved in “better” combination, i.e. combination of residues that have high contact strength, but also strongly weights the number of surrounding residues. The design is consistent with previous findings that catalytic residues are more structurally rigid [19]. For any three residues selected (denoted as *n*, a subset of the *N* surrounding residues), we define an averaged WCN 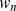, which is the average WCN of these three residues,
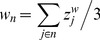
(6)where 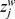 is the normalized WCN, 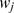, of residue j. Among these three residues, each residue receives a voting score 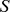,
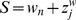
(7)where 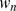 is the averaged WCN and 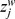 is the normalized WCN of residue j. For each point, the selection process is repeated for all possible combinations of any three surrounding residues. The final score 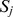 of residue j is the sum of voting scores from all voting scores received,



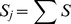
(8)Residues having final score larger than a threshold are predicted as catalytic residue. The threshold for each protein depends on its side chain functional residue number *f*, i.e., the number of residues of amino acid types listed in Table 1. These amino acid types usually have functional atom located on the side chain. According to our observations, the best threshold for each protein depends on its *f*, as in.
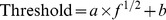
(9)where *a* and *b* are parameters optimized by trying their different combinations and, for each combination, calculating the average MCC of each protein in the PW79 dataset. The combination of *a* and *b* resulting in the highest average MCC was chosen (*a* = 0.06, *b* = 0.88). For protein of larger size, the threshold is more stringent to avoid unnecessary guesses yielding more false positives. Residue that has final score 
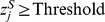
 is predicted as catalytic residue, where 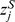 is the normalized *S_j_* of residue *j*.

### Second Phase – Predicting Non-side Chain Functional Amino Acids

Despite most catalytic residues have their functional atom on the side chain; there are a small fraction (approximately 5% in each dataset) of catalytic residues having functional atom on the backbone. For example, the functional atom of most catalysis-related glycine is the backbone amide nitrogen. The second phase is designed to identify these types of amino acids based on the results of the first phase. We pick the top three ranked residues in the final list of first phase and find their neighboring residues with Cα atoms distance less than 10 Å. Note that we only find the neighboring residues with amino acid types that are not listed in Table 1. For each neighboring residue *j*, if its WCN 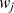 is larger than *w_cut_* = 0.9, residue *j* is predicted to be a catalytic residue.

### Sequence Conservation

The core of the EXIA method is purely based on structure information. It becomes even more powerful by including sequence conservation. The sequence conservation is directly taken from the PSI-Blast [20] position-specific substitution matrix (PSSM) for each protein. PSI-Blast is set to search against the non-redundant (nr) database for three iterations with an E-value threshold of 5×10^−3^. The nr database is a default built-in protein sequence database in PSI-Blast. It includes all non-redundant protein sequences in the GenBank CDS translations, PDB, SwissProt, PIR and PRF. The sequence conservation score *c_j_* of residue *j* is directly taken from the “information per position” column in the PSSM profile. The combination of EXIA and sequence conservation is to directly include *c_j_* in the final score *S_j_* of residue *j* as in,
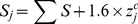
(10)where 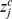 is the normalized *c_j_* of residue *j*.

### Normalization of Scores

The WCN *w_j_*, sequence conservation *c_j_* and the final score *S_j_* of any residue *j*, are normalized to their corresponding z-scores,
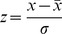
(11)where 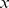 is the original value of *w_j_*, *c_j_* or *S_j_*, 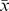 and 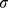 are the average and standard deviation of all corresponding values in the protein. The *normalized w_j_, c_j_* and *S_j_* are denoted as 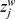, 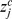 and 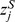 respectively.

### Performance Measurement

The following performance measures are used to evaluate our prediction:

True positive rate (TPR) or recall or sensitivity is denoted as R,



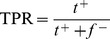
.

False positive rate (FPR),



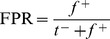
.

Precision (P),



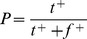
.

Matthew’s correlation coefficient (MCC),
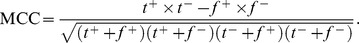
where 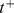, 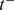, 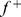, 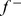 are the true positive, true negative, false positive, and false negative respectively. These measurements are calculated according to the overall performances for each dataset. The receiver operating characteristic (ROC) curve is the average of per-protein ROC curve, which is plotted by changing thresholds from highest (FPR = 0) to lowest (FPR = 1).

### Datasets

Most datasets commonly used in recent competing methods are included here and are constructed based on the data from Catalytic Site Atlas [16]. The datasets include the *PW79* dataset, 79 enzyme selected by [21] and the *POOL160* dataset, 160 enzymes used in [14]. Three datasets with varying homology levels, the *EF fold*, the *EF superfamily* and the *EF family*, used in [22]. The EF datasets are created according to the fold, superfamily, and family levels of SCOP ASTRAL 40 v1.65 [22]. In addition, from the five datasets, we collected all of the structures that do not have any bounded ligands in their crystal structure as the *UB78* dataset. In these datasets, all ligands and non-protein compounds are removed. The full lists of PDB codes and catalytic residues of the six datasets are in the supporting information (Dataset S1, S2, S3, S4, S5, S6). In these datasets, for the proteins missing important side chain atoms (atoms listed in Table 1), we discarded the structure and replaced it with another structure of the same enzyme by manual searching proper replacement with highest sequence and structure similarity as the replaced structure. In few cases that no proper replacement available, we used Cβ atom as side chain vector atom for the structurally incomplete residues. The details are noted in the dataset lists (Dataset S1, S2, S3, S4, S5, S6) in the supporting information.

## Results and Discussion

In this section, we first compared the prediction results of EXIA with other prediction methods that only use structure or sequence information. Then we compared the results of EXIA combined with PSSM with that of the state-of-the-art prediction method. We also discuss the success of EXIA by analyzing side chain orientations of catalytic and non-catalytic residues. In the end, we show the prediction results on enzymes of single catalytic residue and the predictions results on a dataset of enzyme structures intrinsically without any bounded ligand.

### Comparison of EXIA with Predictions using Only Structure Features

To evaluate the performance of the EXIA method, we compared the prediction results using EXIA *without* sequence conservation with the most recent and successful structure-based prediction method, the Partial Order Optimum Likelihood (POOL) method [14], which achieved the best performance among the methods using structure information only. There are two primary structural features in the POOL method: the THEMATICS feature (denoted as POOL(T)) and the cleft size feature (POOL(G)), which is the computational geometry to define and measure pockets on the protein surface using CASTp. Table 2 compares the prediction results of EXIA without sequence conservation with that of POOL with different combinations of features, including POOL(T), POOL(G), and sequence conservation (POOL(C)) for the POOL160 dataset. The results show that EXIA performed obviously better than POOL(T+G) on recall at equal precision, precision at equal recall, and area under curve receiver operating characteristic (AUCROC). POOL achieved the best performance combining POOL(T), POOL(G), and POOL(C). EXIA, even without using sequence conservation, performed significantly better than POOL, which used both sequence and structure information. The comparison of ROC curves of EXIA and POOL on the POOL160 dataset is shown in figure S2.

**Table 2:** Comparison of EXIA prediction with POOL on POOL160 dataset.

	POOL using different features	
	POOL(T+G)^1^	POOL(T+G+C)^2^
Recall	61.74	64.68
Precision	18.06	19.07
AUCROC	0.907	0.925
	**EXIA without PSSM**	
Recall at equal P	70.80^3^	68.60^5^
Precision at equal R	22.20^4^	20.80^6^
AUCROC	0.960	0.960
	**EXIA+PSSM**	
Recall at equal P	80.00	77.80
Precision at equal R	24.30	23.30
AUCROC	0.969	0.969

1 POOL method using only structure features (POOL(T): THEMATICS and POOL(G): geometry features).

2 POOL method using sequence (POOL(C): sequence and sequence conservation) and structure features.

3 Recall of EXIA at equal precision as POOL(T+G).

4 Precision of EXIA at equal recall as POOL(T+G).

5 Recall of EXIA at equal precision as POOL(T+G+C).

6 Precision of EXIA at equal recall as POOL(T+G+C).

### Comparison of EXIA with Predictions using Only Sequence-based Features

In the development of catalytic residues prediction methods, sequence information was the primary feature, which was usually based on information of amino acid types and sequence conservation. Incorporation of structure information did improve the results of sequence-based prediction, but predictions using *only* structure information had never been found to be comparable to sequence-based predictions. These structure information used to predict catalytic residues includes experimental or computational backbone flexibility, relative solvent accessible surface area, atomic density, physical and chemical properties in 3D environments, cleft shape and size [21], [23], network centrality [24], and etc. According to previous reports [21], [23], the prediction accuracies based on individual one of these structure features were mostly from 51% to 60% except predictions based on well-designed cleft shape and hydrogen bonding number statistics, which have accuracies from 63% to 69%. However, prediction using only amino acid type information could easily reach a prediction accuracy of 70% and using only sequence conservation has over 80% accuracy.

Here we directly compared EXIA *without* sequence conservation with the state-of-the-art sequence-based prediction method, CRpred [25], which was shown to have comparable results with predictions using both structure and sequence information. The novelty of CRpred is the design of several new types of sequence-based features computed using windowed hydrophobicity, custom-designed sequence motifs, and position-specific scoring matrix and entropy of weighted observed percentages from PSI-BLAST. Table 3 summarizes the prediction results of CRpred and EXIA without PSSM on four benchmark datasets. EXIA outperforms CRpred on the PW79 dataset by comparing the recall at equal precision and the precision at equal recall. EXIA has recall (0.68) and precision (0.25) higher than theirs (R = 0.54, P = 0.18). On the EF fold and EF superfamily datasets, EXIA has comparable results to CRpred. On the EF family dataset, CRpred has better prediction results than EXIA. It is interesting to note that CRpred has best prediction results on the EF family dataset, which has higher homology level. On the datasets that have lower homology levels, the EF fold and EF superfamily datasets, the performances of CRpred slightly decreases. EXIA performs equally well on these three datasets with AUCROC from 0.940 to 0.944 without being affected by the differences of homology levels. The comparison of ROC curves of EXIA and CRpred on the EF fold dataset is shown in figure S1.

**Table 3:** Comparison of EXIA prediction with CRpred on four benchmark datasets.

	Benchmark datasets			
	PW79	EF fold	EF superfamily	EF family
**CRpred**				
Recall (R)	53.7	48.2	52.1	58.3
Precision (P)	17.5	17.0	17.0	18.6
**EXIA** 1				
Recall at equal P	67.8	45.1	49.5	45.8
Precision at equal R	24.7	16.2	16.1	14.6
AUCROC	0.961	0.940	0.940	0.944

1 Prediction results using EXIA without sequence conservation.

The point of this comparison is not to determine whether structure information is more important or efficient than sequence information. Structure and sequence information are both important and are complementary features in catalytic residue prediction. EXIA is the first pure structure-based method that has comparable prediction performances to sequence-based predictions. Another advantage of EXIA is that it only requires single protein structure without needing sequence or structure comparisons, which are usually required in sequence-based methods. In the next section, we combined EXIA and sequence conservation from PSI-Blast PSSM profiles.

### Combination of EXIA and Sequence Conservation and Comparison with State-of-the-art Method

A recent prediction method [15] outperformed other existing methods on various benchmark datasets. Their method is based on effective representation of structure information by modeling spherical regions around candidate residues and statistics on the physic-chemical and structural properties in the region. They used support vector machine to predict catalytic residues based on these features combined with sequence information and made a wide and complete comparisons with other competing methods. Table 4 summarizes the prediction results of EXIA combined with PSSM (EXIA+PSSM) and comparison with their results on five datasets. We compared the best recall and precision values in their report with our recall at equal precision and our precision at equal recall. EXIA+PSSM has higher recall and precision than theirs in most comparisons except in the POOL160 dataset. The precision (0.189) and recall (0.780) are almost equal to theirs (0.190 and 0.781). By comparing AUCROC (area under ROC curve), which is a more reliable and global measure of performance, EXIA+PSSM outperforms the competing method on both PW79 and POOL160 datasets. Figure S3 also shows the comparison of ROC curves on the EF fold dataset.

**Table 4:** Comparison of EXIA prediction with competing methods on five benchmark datasets.

	Benchmark datasets				
	PW79	POOL160	EF fold	EF superfamily	EF family
**Competing method** 1					
Recall (R)	46.0	78.1	64.2	67.3	61.7
Precision (P)	28.0	19.0	17.1	16.9	18.5
AUCROC	0.963	0.948	–	–	–
**EXIA+PSSM** 2					
Recall at equal P	63.0	78.0	72.3	72.4	69.0
Precision at equal R	34.7	18.9	20.2	18.9	21.1
AUCROC	0.978	0.969	0.968	0.965	0.966
**EXIA without PSSM** 3					
Recall at equal P	48.9	68.6	44.8	50.0	46.3
Precision at equal R	30.3	14.4	12.0	11.9	13.7
AUCROC	0.962	0.960	0.940	0.940	0.944

1 Prediction results by Cilla and Passerini [15].

2 Prediction using EXIA combined with sequence conservation.

3 Prediction using EXIA without sequence conservation.

### Prediction Results of Combining EXIA and Sequence Conservation

**Figure 3:**
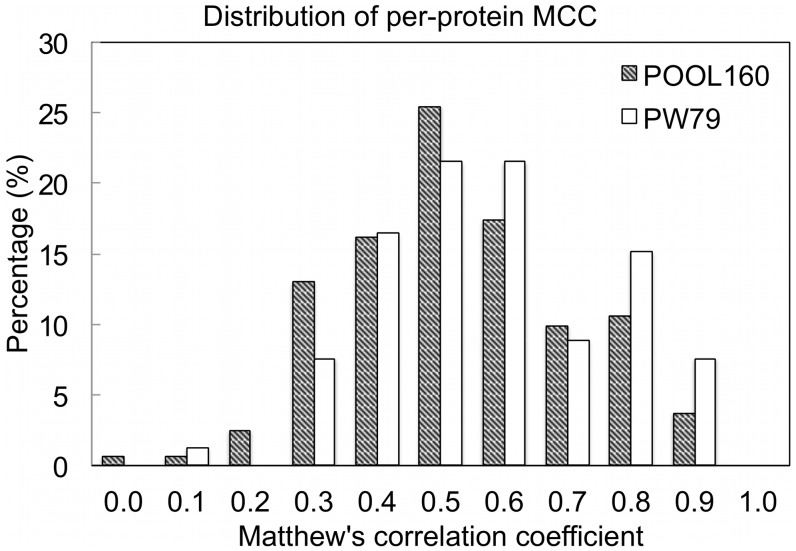
Distributions of Matthew’s correlation coefficient for each protein in the PW79 and POOL160 datasets. The per-protein Matthew’s correlation coefficient of prediction combined EXIA and sequence conservation. MCC was calculated without changing the ratio of catalytic and noncatalytic residues in the datasets.

Matthew’s correlation coefficient (MCC) is a good measurement of prediction performance because that MCC is very sensitive to false positives. Due to the extremely unbalanced number of catalytic and non-catalytic residues in enzymes, MCC were in the range of 0.23 to 0.36 for the PW79 dataset in previous predictions [15], [21] because of the large number of possible false positives. The unbalanced number of catalytic and non-catalytic residues also causes problems in machine learning method, for example, model training in support vector machine. To avoid such problem, a commonly used strategy is to build a *balanced* dataset in which the ratio between catalytic and non-catalytic residues is equal by subsampling non-catalytic residues [21], [23]. When the subsampling strategy is applied to testing dataset, MCC increases greatly to 0.7∼0.8 because that the number of possible false positives (non-catalytic residues) is greatly reduced [21]. Here, MCC was calculated without changing the ratio of catalytic and non-catalytic residues. Figure 3 shows the MCC of EXIA prediction for each protein in the POOL160 and PW79 datasets. For the POOL160 dataset, there are 42% of proteins having 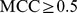 and the average MCC is 0.48. For the PW79 dataset, there are 53% of proteins having 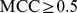 and the average MCC is 0.53.

**Figure 4:**
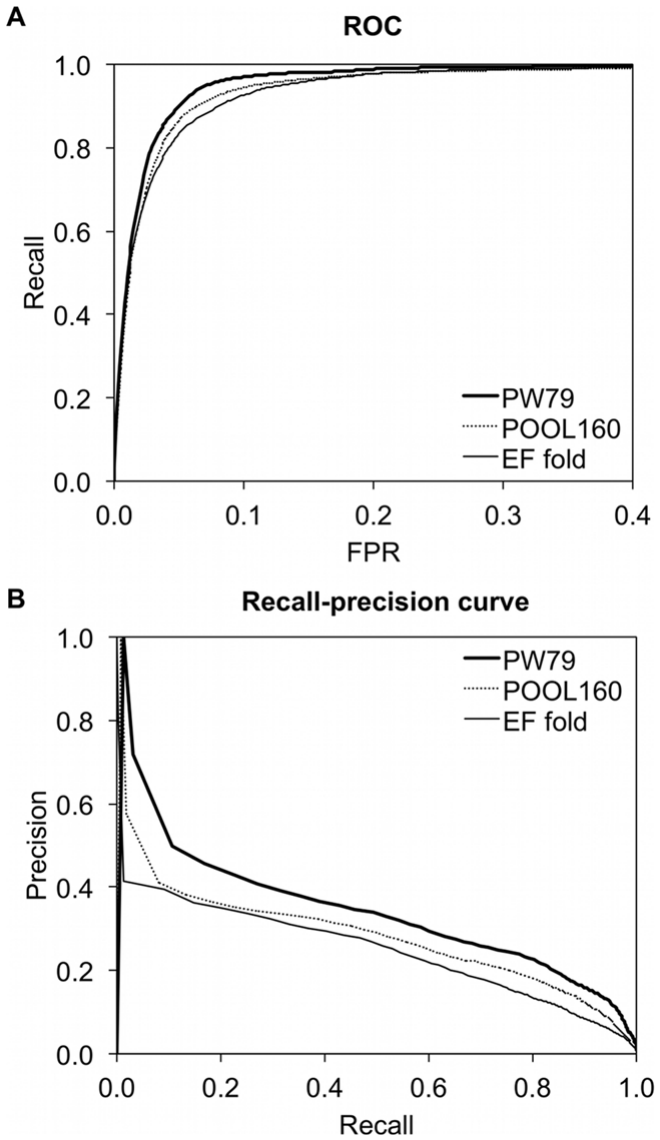
ROC and Recall-precision curves of EXIA+PSSM on the PW79, POOL160, and EF fold datasets. (A) ROC and (B) Recall-precision curves of EXIA+PSSM on the PW79, POOL160, and EF fold datasets. EF superfamily and EF family datasets had similar results to the EF fold dataset.

Figure 4 shows the overall ROC and Recall-precision (RP) curves of EXIA+PSSM on the benchmark datasets. The ROC and RP curves on EF superfamily and EF family datasets are very similar to that of EF fold dataset and are not shown in the figure.

**Figure 5:**
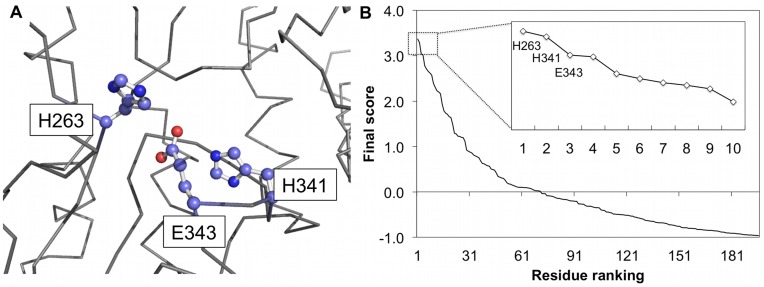
Structures of catalytic residues of human ferrochelatase and results of prediction. (A) Structures of catalytic residues, H263, H341, and E343, of human ferrochelatase (PDB id: 1 hrk). Side chain structures of the catalytic residues are shown as ball-and-stick. (B) Prediction results of human ferrochelatase by EXIA without sequence conservation. This figure shows the distribution of ranked final score of residues. The catalytic residues, H263, H341, and E343, are the top three ranked residues.

Figure 5 shows the structures of catalytic residues and prediction results of a typical example, human ferrochelatase (PDB id: 1 hrk), which is a homodimer that catalyzes the insertion of ferrous iron into protoporphyrin to form heme. The side chain orientations of catalytic residues, H263, H341, and E343, on its A chain are shown in figure 5(A). Figure 5(B) is the prediction results of the enzyme by EXIA without using sequence conservation and based on the isolated A chain structure. The figure shows the ranked final score 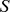 of residues and the catalytic residues are the top three ranked ones.

### Why does EXIA Work? Analysis on Side Chain Orientations of Catalytic Residues

**Figure 6:**
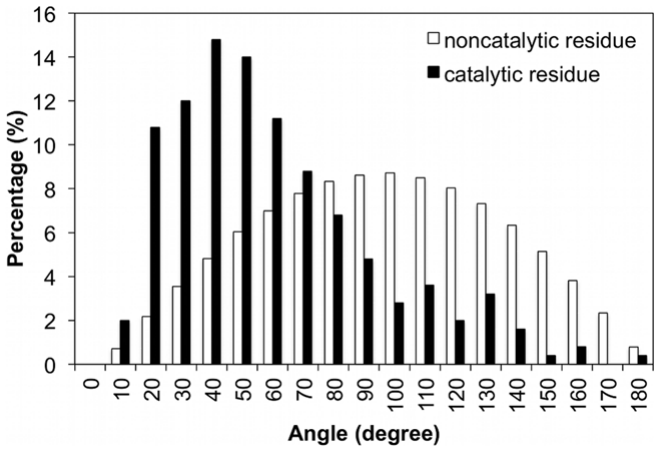
Comparison of side chain orientation of catalytic residues and randomly selected residues. The side chain orientation of catalytic residues is obviously different from those of random residues. Smaller angle means that residue side chain tends to point to the centroid of the residue group (details in the corresponding section). The angles for catalytic residues are obvious smaller than random selected residues.

The design of EXIA method is based on the special orientation of catalytic residues in enzyme. Here, we directly compared the side chain orientations of catalytic residues with that of randomly selected residues. For each enzyme in the PW79 dataset (except enzymes of single catalytic residue), we calculated the center-of-mass of the catalytic residues and assumed the center-of-mass is approximately the center of the “catalytic spot”. First, for each catalytic residue *j*, we calculated the angle between the side chain vector of residue *j* and the vector from Cα atom of residue *j* to the center-of-mass position. Second, for randomly selected residues, each protein in the dataset was embedded in a 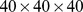 grid of points. For each point, we found its neighboring residues (distance between the point and the residue <10 Å) and repeated the angle calculation for this group of residues (as a group of spatially close residues randomly picked). Figure 6 compares the angles for catalytic residues with angles for random residues. The range of angles are originally between −180° to 180°, we converted the angle values to its corresponding absolute value. The results are obvious that the angle distribution for randomly picked residues is a normal distribution from 0° to 180°, which means that for a randomly chosen spot in protein structure, the side chain orientations of residues surrounding the spot are random. Side chains for random residues do not seem to point to any particular position. For catalytic residues, the angles are significantly smaller than those of random residues (statistically significant tested by a paired t-test with α = 0.001). The results suggest that catalytic residues do have very special side chain orientation comparing to random groups of residues in protein. The side chain vector of catalytic residue tends to point to the center of the catalytic spot, which is approximated by the center-of-mass of catalytic residues. The special orientation of side chain is a unique structure feature of catalytic residues and is the foundation of success of the EXIA method.

**Figure 7:**
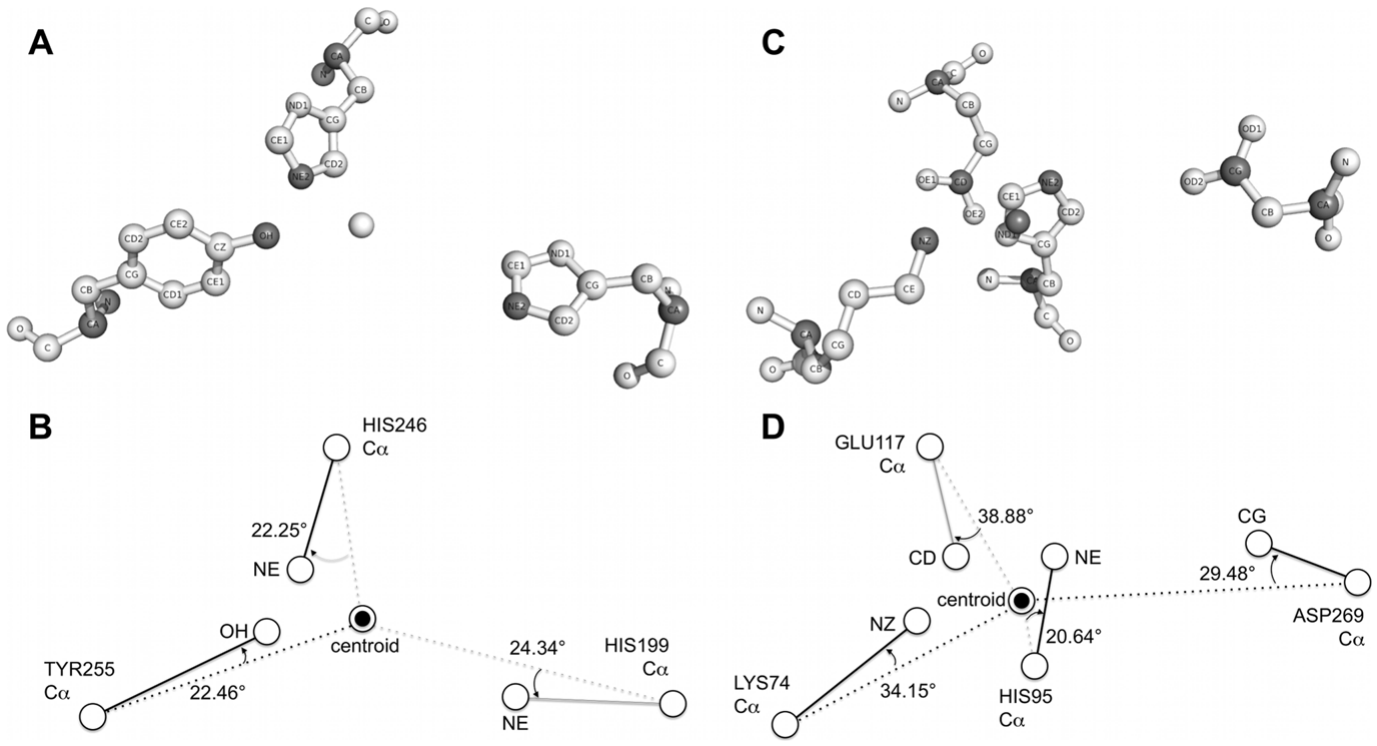
Side chain orientation of catalytic residues in two example enzymes. The catalytic residue structures of (A) metapyrocatechase from *Ppseudomonas putida mt-2* (PDB id: 1mpy) and (C) L-alanine dehydrogenase (PDB id: 1pjb). Atoms colored in dark grey are the Cα atom and side chain vector atom for each residue. (B) and (D) illustrate the position of center-of-mass of these catalytic residues (centroid) and their side chain vector. These examples clearly show that the side chain vectors tend to point to the centroid.

Figure 7 shows the catalytic residue structures of metapyrocatechase from *Ppseudomonas putida mt-2* (PDB id: 1mpy) and L-alanine dehydrogenase (PDB id: 1pjb). In figure 7(A) and (C), atoms colored in dark grey are the Cα atoms and side chain vector atoms. Figure 7(B) and (D) illustrate the position of the center-of-mass of these catalytic residues and their side chain vectors. In metapyrocatechase, the side chains of catalytic residues, H199, H246, and Y255, all point to their center-of-mass. The same phenomenon is observed on the catalytic residues, K74, H95, E117, and D269 of L-alanine dehydrogenase. It is not possible to observe that side chain of catalytic residues point to the center-of-mass perfectly. The side chain orientation of catalytic residues must properly “fit” the ligand it catalyzed. The results in figure 6 still clearly show that side chains of catalytic residues tend to point to their center-of-mass.

### Comparison of Prediction Results on Residue Level

In the reports of these competing methods, there are not much residue-level prediction results available for direct comparison. The POOL method is the only one that provides a web server [26]. We have manually submitted five proteins, including dimerization cofactor of hepatocyte nuclear factor 1 (PDB id: 1dco), UDP N-acetylglucosamine acyltransferase (1lxa), catechol 2,3-dioxygenase (1mpy), uucleoside diphosphate kinase (1nsp), and acylphosphatase (2acy), to the web server. We compared the prediction rank of EXIA+PSSM and POOL for the catalytic residues of each protein. Table 5 summarizes the results and the number of false positives when all true positives (catalytic residues) are correctly identified. In these examples, EXIA performs better than POOL, having catalytic residues highly ranked and correctly identifying them with smaller number of false positives than those of POOL.

**Table 5:** Comparison of prediction rank and number of false positives for EXIA+PSSM and POOL.

Protein	Catalytic residue	Prediction rank		Number of false positives when all catalytic residues are identified	
		EXIA+PSSM	POOL	EXIA+PSSM	POOL
1dco	H62	2	1	0	5
	H63	3	5		
	H80	4	9		
	D89	1	4		
1lxa	H125	2	4	1	3
1mpy	H199	2	6	3	4
	H246	6	7		
	Y255	1	1		
1nsp	K16	4	4	4	18
	N199	7	21		
	H122	1	10		
2acy	R23	3	8	1	7
	N41	2	9		

### Prediction Performance of Enzymes of Single Catalytic Residue

A small fraction of enzymes in these benchmark datasets (10% ∼ 20%) have only one catalytic residue. Although the basic concept of EXIA method is to find multiple residues that have their side chains pointing to a certain point, for proteins having single catalytic residues, EXIA is still able to identify their catalytic residue. Table 6 summarizes the prediction results of single-catalytic-residue proteins on the five datasets. The average MCC of single-catalytic-residue proteins on PW79 and POOL160 datasets are 0.44 and 0.36, which are lower than the overall average MCC on these two datasets (0.53 and 0.48 respectively) but are higher than the best MCC reported before [15]. The reason is that EXIA is based on not only side chain orientations but also the backbone flexibility, which is highly related to catalytic residues. It was found that catalytic residues are usually structurally rigid [19], harder to be moved [12], or have high closeness centrality in network of protein structure [24]. The design of EXIA includes backbone flexibility in the voting scores based on the weighted-contact number model. Another reason may be that, even for single-catalytic-residue proteins, their catalytic residue tends to locate in such environment, i.e. side chain orientations identified by EXIA.

**Table 6:** Average MCC of proteins having single catalytic residue on the five datasets.

		Average MCC	
Benchmark datasets	Number of proteins^1^	EXIA+PSSM^2^	EXIA^3^
PW79	12	0.44	0.45
POOL160	16	0.36	0.42
EF fold	36	0.40	0.30
EF superfamily	44	0.40	0.33
EF family	55	0.38	0.33

1 Number of proteins having single catalytic residue.

2 Prediction using combination of EXIA and sequence conservation.

3 Prediction using EXIA.

### Prediction Performance on Unbound Structures

A dataset (UB78), which includes all structures that have no bounded ligand in their crystal structure from the PW79, POOL160, and three EF datasets, is used to test the performance of EXIA on unbound enzyme structures. The overall AUCROC of EXIA+PSSM prediction on the UB78 dataset is 0.961, which is similar to the results on the POOL160 (0.969), EF_fold (0.968), EF_superfamily (0.965), and EF_family (0.966) datasets. The overall AUCROC of EXIA without PSSM prediction on the UB80 dataset is 0.941, which is also similar to the results on the EF_fold (0.940), EF_superfamily (0.940), and EF_family (0.944) datasets. The results show that EXIA works well in the unbound structures too. The results also suggest that the side chains of catalytic residues, even in the unbound state, are ready to form a catalytic spot to interact with ligand and are distinct from other non-catalytic residues.

### Conclusions

We found that catalytic residues in enzyme have very special orientation of side chain comparing to those of random residues. Based on the novel observation that the side chain of catalytic residues usually points to the center of catalytic spot, we have developed a purely structure-based method, EXIA, to predict catalytic residues EXIA identifies catalytic residues by finding residues with such property and the prediction results show that catalytic residues can be correctly predicted from protein structure in various benchmark datasets, including a dataset of ligand unbound structures.

Structure information is usually thought to be more “informative” than sequence information in bioinformatics studies of proteins. On the contrary, sequence information were more effective features than structure information in prediction of protein catalytic residues. Prediction method using only sequence information was shown to perform almost equally well to methods using both sequence and structure information [25]. We show that EXIA is currently the most effective structure-based method and, without using any sequence information, is comparable to the state-of-the-art sequence-based method. The prediction of combining EXIA and sequence information outperforms existing prediction methods and has average MCC from 0.48 to 0.53 on five benchmark datasets.

A recent study found that evolutionary information is actually hidden in single protein structure [27]. The backbone flexibility profile computed from single protein structure and its PSSM profile from PSI-Blast are found to be quite similar. The success of EXIA also suggests that information properly extracted from protein structure is very powerful in the prediction of catalytic residues. For proteins whose evolutionary information is not available, EXIA is still able to provide invaluable information in the study of protein functions.

## Supporting Information

Figure S1 **ROC curves of EXIA and CRpred on the EF fold dataset.**
(PDF)

Figure S2 **ROC curves of EXIA and POOL on the POOL160 dataset.**
(PDF)

Figure S3 **ROC curves of EXIA and results by Cilla and Passerini on the EF fold dataset.**
(PDF)

Dataset S1 List of PDB for the PW79 dataset.
(DOCX)

Dataset S2 List of PDB for the POOL160 dataset.
(DOCX)

Dataset S3 List of PDB for the EF fold dataset.
(DOCX)

Dataset S4 List of PDB for the EF superfamily dataset.
(DOCX)

Dataset S5 List of PDB for the EF family dataset.
(DOCX)

Dataset S6 List of PDB for the UB78 dataset.
(DOCX)
