# Supplementary figures and images for: Accurate Prediction of Protein Catalytic Residues by Side Chain Orientation and Residue Contact Density
Source: PLoS One. 2012 Oct 24;7(10):e47951. doi: 10.1371/journal.pone.0047951 (PMC3480458; doi:10.1371/journal.pone.0047951)

Fig. S1. ROC curves of EXIA and CRpred on the EF fold dataset

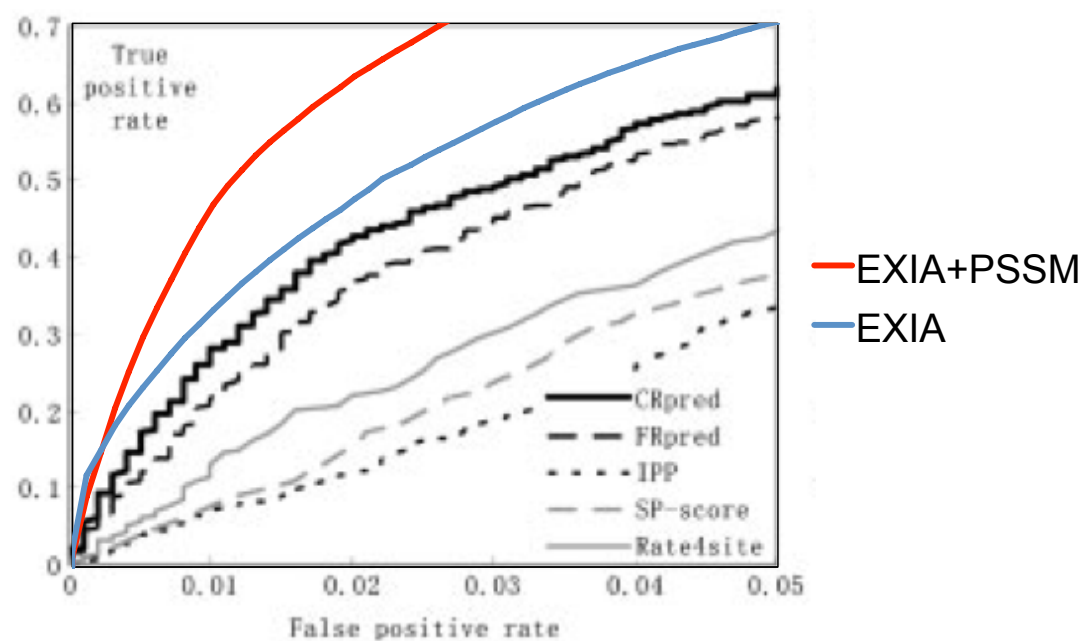

Supplement: Figure S1 — ROC curves of EXIA and CRpred on the EF fold dataset. (PDF) [file pone.0047951.s001.pdf]

Fig. S2. ROC curves of EXIA and POOL on the POOL160 dataset

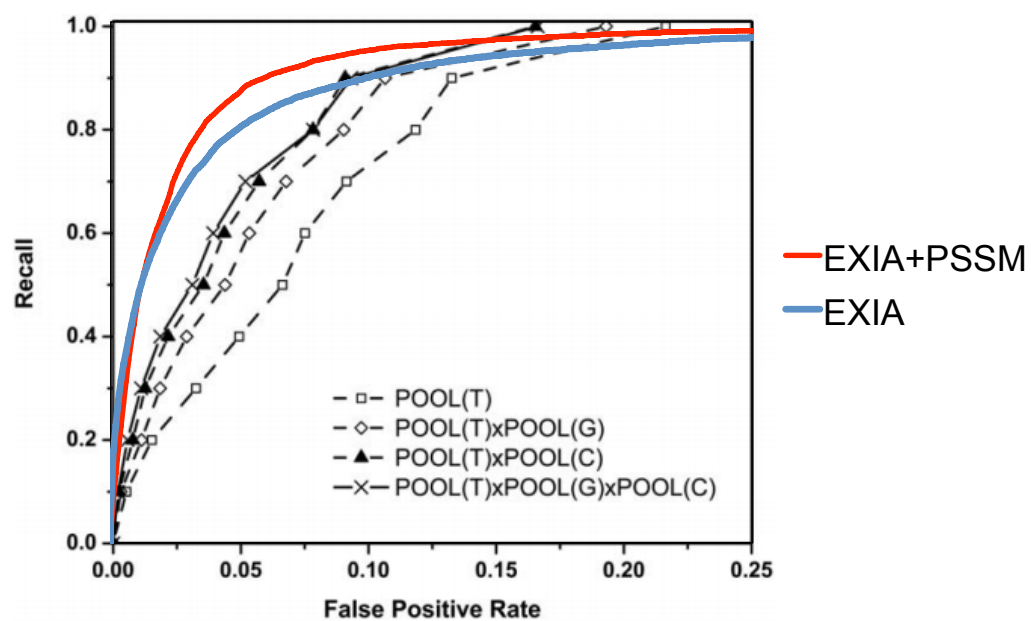

Supplement: Figure S2 — ROC curves of EXIA and POOL on the POOL160 dataset. (PDF) [file pone.0047951.s002.pdf]

Fig. S3. ROC curves of EXIA and results by Cilla and Passerini on the EF fold dataset

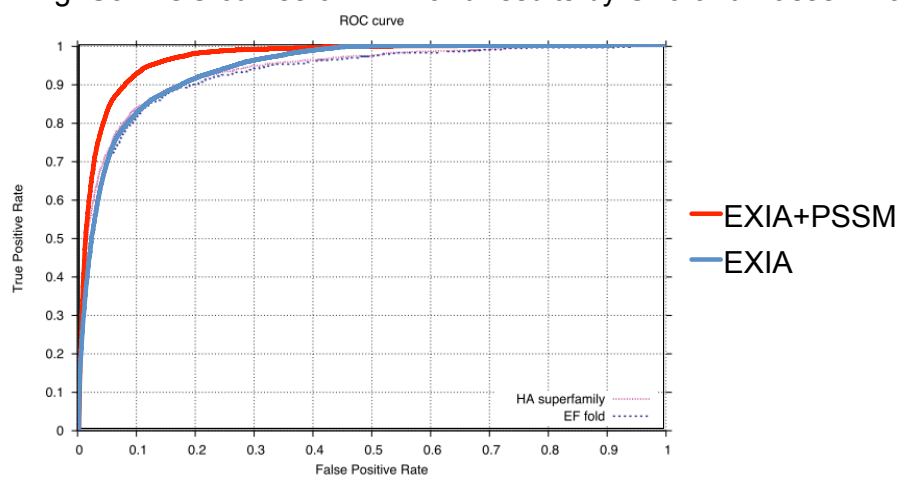

Supplement: Figure S3 — ROC curves of EXIA and results by Cilla and Passerini on the EF fold dataset. (PDF) [file pone.0047951.s003.pdf]
