# Supplementary material for: Accurate Prediction of Protein Catalytic Residues by Side Chain Orientation and Residue Contact Density
Source: PLoS One. 2012 Oct 24;7(10):e47951. doi: 10.1371/journal.pone.0047951 (PMC3480458; doi:10.1371/journal.pone.0047951)
Supplement: Dataset S1 — List of PDB for the PW79 dataset. (DOCX) [file pone.0047951.s004.docx]

Dataset S1: PW79

| **PDB** | **Chain** | **CSA Annotated Active Site Residues** | **Note** |
| --- | --- | --- | --- |
| 1a26 | A | TYR907, GLU988 |  |
| 1a4i | A | LYS56, |  |
| 1a4s | A | ASN166, GLU263, CYS297, |  |
| 1ab8 | A | ARG1029, | Replaced by 1culB |
| 1ae7 | A | GLY30, HIS48, ASP99, |  |
| 1afw | A | CYS125, HIS375, CYS403, GLY405 |  |
| 1ah7 | A | ASP55, |  |
| 1akm | A | ARG106, HIS133, GLN136, ASP231, CYS273, ARG319 |  |
| 1aop | A | ARG83, ARG153, LYS215, LYS217, CYS483, |  |
| 1apx | A | ARG38, HIS42, ASN71, |  |
| 1apy | B | THR183, THR201, THR234, GLY235 |  |
| 1aq2 | A | HIS232, LYS254, ARG333, |  |
| 1aw8 | B | TYR58, |  |
| 1b3r | A | ASP130, LYS185, ASP189, ASN190, CYS194, |  |
| 1b57 | A | ASP109, GLU182, ASN286, | Replaced by 1dosA |
| 1b93 | A | HIS19, GLY66, ASP71, ASP91, HIS98, ASP101 |  |
| 1bol | A | HIS46, GLU105, HIS109, |  |
| 1brm | A | CYS135, GLN162, HIS274, |  |
| 1bs4 | A | GLY45, GLN50, LEU91, GLU133 |  |
| 1btl | A | SER70, LYS73, SER130, GLU166 |  |
| 1bzy | A | GLU133, ASP134, ASP137, LYS165, ARG169, |  |
| 1cd5 | A | ASP72, ASP141, HIS143, GLU148 |  |
| 1chd | A | SER164, THR165, HIS190, MET283, ASP286, |  |
| 1ctt | A | GLU104, |  |
| 1d4a | A | GLY149, TYR155, HIS161, |  |
| 1daa | A | LYS145, GLU177, LEU201, |  |
| 1dae | A | THR11, LYS15, LYS37, SER41 |  |
| 1db3 | A | THR132, GLU134, TYR156, LYS160 |  |
| 1dbt | A | ASP60, LYS62 |  |
| 1dco | C | HIS62, HIS63, HIS80, ASP89 |  |
| 1diz | A | TYR222, ASP238, TRP272, |  |
| 1dj0 | A | ASP60, |  |
| 1dnk | A | GLU78, HIS134, ASP212, HIS252 |  |
| 1dnp | A | TRP306, TRP359, TRP382, |  |
| 1dqs | A | HIS275, |  |
| 1dzr | A | HIS63, ASP170 |  |
| 1e2a | A | HIS78, GLN80, ASP81, HIS82 |  |
| 1ef8 | A | HIS66, GLY110, TYR140, |  |
| 1eyi | A | ASP68, ASP74, GLU98, |  |
| 1fua | A | GLU73, |  |
| 1gim | A | ASP13, HIS41, GLN224, |  |
| 1gpm | A | GLY59, CYS86, TYR87, HIS181, GLU183, ASP239 |  |
| 1gpr | A | THR66, HIS68, HIS83, GLY85 |  |
| 1grc | A | ASN106, HIS108, SER135, ASP144 | Replaced by 1cddA |
| 1hxq | A | CYS160, HIS164, HIS166, GLN168 |  |
| 1iph | A | HIS128, SER167, ASN201, |  |
| 1jdw | A | ASP254, HIS303, CYS407, |  |
| 1kas | A | CYS163, HIS303, HIS340, PHE400 |  |
| 1kra | C | HIS219, ASP221, HIS320, ARG336 |  |
| 1lba | A | TYR46, LYS128 |  |
| 1lxa | A | HIS125, |  |
| 1mbb | A | ARG159, SER229, GLU325, |  |
| 1mek | A | CYS36, GLY37, HIS38, CYS39 |  |
| 1mla | A | SER92, HIS201, GLN250, |  |
| 1moq | A | GLU481, LYS485, GLU488, HIS504, LYS603, |  |
| 1mpy | A | HIS199, HIS246, TYR255, |  |
| 1nba | A | ASP51, LYS144, ALA172, THR173, CYS177, |  |
| 1nsp | A | LYS16, ASN119, HIS122, |  |
| 1pfk | A | GLY11, ARG72, THR125, ASP127, ARG171, |  |
| 1pjb | A | LYS74, HIS95, GLU117, ASP269 |  |
| 1pnl | B | SER1, ALA69, ASN241, |  |
| 1pud | A | ASP102, |  |
| 1qfe | A | GLU86, HIS143, LYS170, |  |
| 1smn | A | ARG87, HIS89, ASN119, | Replaced by 1qaeA |
| 1uae | A | ASN23, CYS115, ASP305, ARG397 |  |
| 1ula | A | HIS86, GLU89, ASN243, |  |
| 1uok | A | ASP199, GLU255, ASP329, |  |
| 1uox | A | ARG176, GLN228 |  |
| 1wgi | A | ASP117, | Replaced by 1hukA |
| 1xva | A | GLU15, |  |
| 2acy | A | ARG23, ASN41 |  |
| 2alr | A | TYR49, LYS79 |  |
| 2bbk | L | ASP32, TRP57, ASP76, TRP108, TYR119, THR122 |  |
| 2cpo | A | HIS105, GLU183 |  |
| 2hgs | A | ARG125, SER151, GLY369, ARG450 |  |
| 2jcw | A | HIS63, ARG143 |  |
| 2pfl | A | TRP333, CYS418, CYS419, GLY734 |  |
| 2plc | A | HIS45, ASP46, ARG84, HIS93, ASP278, |  |
| 3eca | A | THR12, TYR25, THR89, ASP90, LYS162, |  |
