# Supplementary material for: Accurate Prediction of Protein Catalytic Residues by Side Chain Orientation and Residue Contact Density
Source: PLoS One. 2012 Oct 24;7(10):e47951. doi: 10.1371/journal.pone.0047951 (PMC3480458; doi:10.1371/journal.pone.0047951)
Supplement: Dataset S2 — List of PDB for the POOL160 dataset. (DOCX) [file pone.0047951.s005.docx]

Dataset S2: POOL160

| **PDB** | **Chain** | **CSA Annotated Active Site Residues** | **Note** |
| --- | --- | --- | --- |
| 12as | A | ASP46, ARG100, GLN116 |  |
| 13pk | A | ARG39, LYS219, GLY376, GLY399, |  |
| 1a05 | AB | ATYR140, BLYS190, BASP222 |  |
| 1a26 | A | TYR907, GLU988, |  |
| 1a4i | A | LYS56 |  |
| 1a4s | A | ASN166, GLU263, CYS297 |  |
| 1ae7 | A | GLY30, HIS48, ASP99 |  |
| 1afw | A | CYS125, HIS375, CYS403, GLY405, |  |
| 1ah7 | A | ASP55 |  |
| 1akm | A | ARG106, HIS133, GLN136, ASP231, CYS273, ARG319, |  |
| 1alk | A | SER102, ARG166, |  |
| 1aop | A | ARG83, ARG153, LYS215, LYS217, CYS483 |  |
| 1apx | A | ARG38, HIS42, ASN71 |  |
| 1apy | B | THR183, THR201, THR234, GLY235, |  |
| 1aq2 | A | HIS232, LYS254, ARG333 |  |
| 1aw8 | B | TYR58 |  |
| 1b3r | A | ASP130, LYS185, ASP189, ASN190, CYS194 |  |
| 1b57 | A | ASP109, GLU182, ASN286 | Replaced by 1dosA |
| 1b66 | A | ACYS42, BASP88, BHIS89, AGLU133, | Replaced by 1gtqA |
| 1b6b | A | SER97, LEU111, HIS122, LEU124, TYR168 |  |
| 1b73 | A | ASP7, SER8, CYS70, CYS178, |  |
| 1b93 | A | HIS19, GLY66, ASP71, ASP91, HIS98, ASP101, |  |
| 1bcr | A | AGLY53, ASER146, ATYR147, BASP338, BHIS397 | Replaced by 3sc2A |
| 1bg0 | A | ARG126, GLU225, ARG229, ARG280, ARG309 |  |
| 1bjp | A | APRO1, APHE50 |  |
| 1bml | A | HIS603, ASP646, |  |
| 1bol | A | HIS46, GLU105, HIS109 |  |
| 1brm | A | CYS135, GLN162, HIS274 |  |
| 1brw | A | HIS82, ARG168, SER183, LYS187, |  |
| 1bs4 | A | GLY45, GLN50, LEU91, GLU133, |  |
| 1btl | A | SER70, LYS73, SER130, GLU166, |  |
| 1bwd | A | ASP108, ARG127, ASP179, HIS227, ASP229, HIS331, CYS332 |  |
| 1bwp | A | SER47, GLY74, ASN104, ASP192, HIS195 |  |
| 1bzy | A | GLU133, ASP134, ASP137, LYS165, ARG169 |  |
| 1c3c | A | AHIS141, BGLU275, | Replaced by 2x75A |
| 1c3j | A | GLU22, ASP100, |  |
| 1cb8 | A | HIS225, TYR234, ARG288 |  |
| 1cd5 | A | ASP72, ASP141, HIS143, GLU148, |  |
| 1chd | A | SER164, THR165, HIS190, MET283, ASP286 |  |
| 1chk | A | GLU22, ASP40, |  |
| 1chm | A | HIS232, GLU262, GLU358 |  |
| 1coy | A | GLU361, HIS447, ASN485 |  |
| 1cqq | A | HIS40, GLU71, GLY145, CYS147, |  |
| 1ctt | A | GLU104 |  |
| 1d0s | A | GLU317 |  |
| 1d4a | A | GLY149, TYR155, HIS161 |  |
| 1d4c | A | HIS364, ARG401, HIS503, ARG544, |  |
| 1d8c | A | ASP270, GLU272, ARG338, ASP631, | Replaced by 2jqxA |
| 1d8h | A | ARG393, GLU433, LYS456, ARG458, | Replaced by 3kyhA. Use atom CB as side chain vector atom for residue D438 and D440 |
| 1daa | A | LYS145, GLU177, LEU201 |  |
| 1dae | A | THR11, LYS15, LYS37, SER41, |  |
| 1db3 | A | THR132, GLU134, TYR156, LYS160, |  |
| 1dbt | A | ASP60, LYS62, |  |
| 1dco | C | HIS62, HIS63, HIS80, ASP89, |  |
| 1dgs | A | LYS116, ASP118, ARG196, LYS312, |  |
| 1dii | A | TYR95, GLU380, GLU427, HIS436, ARG474 |  |
| 1diz | A | TYR222, TRP272, ASP238 |  |
| 1dl2 | A | GLU132, ARG136, ASP275, GLU435, | Use atom CB as side chain vector atom for residue R75, K409 and K539 |
| 1dli | A | THR118, GLU145, LYS204, ASN208, CYS260, ASP264, |  |
| 1dnk | A | GLU78, HIS134, ASP212, HIS252, |  |
| 1do8 | A | TYR112, LYS183, ASP278 |  |
| 1dqs | A | HIS275 |  |
| 1dzr | A | HIS63, ASP170, |  |
| 1e2a | A | HIS78, GLN80, ASP81, HIS82, |  |
| 1ebf | A | ASP219, LYS223, |  |
| 1ef8 | A | HIS66, GLY110, TYR140 |  |
| 1eug | A | ASP64, HIS187, |  |
| 1eyi | A | ASP68, ASP74, GLU98 |  |
| 1fgh | A | ASP100, HIS101, HIS147, ASP165, HIS167, GLU262, SER642 |  |
| 1foh | A | ASP54, ARG281, TYR289 |  |
| 1fro | A | GLU172 |  |
| 1fua | A | GLU73 |  |
| 1fug | AB | BHIS14, BLYS165, BARG244, BLYS245, ALYS265, ALYS269, AASP271 |  |
| 1fui | A | GLU337, ASP361, |  |
| 1g72 | A | ASP297 |  |
| 1get | AB | BCYS42, BCYS47, BLYS50, BTYR177, BGLU181, AHIS439, AGLU444 |  |
| 1gim | A | ASP13, HIS41, GLN224 |  |
| 1gog | A | CYS228, TYR272, TRP290, TYR495, |  |
| 1gpm | A | GLY59, CYS86, TYR87, HIS181, GLU183, ASP239, |  |
| 1gpr | A | THR66, HIS68, HIS83, GLY85, |  |
| 1grc | A | ASN106, HIS108, SER135, ASP144, | Replaced by 1cddA |
| 1gtp | A | HIS112, HIS179, |  |
| 1hfs | A | GLU202, MET219, |  |
| 1hxq | A | CYS160, HIS164, HIS166, GLN168, |  |
| 1i7d | A | GLU7, LYS8, PHE328, ARG330, |  |
| 1ivh | A | GLU254 |  |
| 1jdw | A | ASP254, HIS303, CYS407 |  |
| 1kas | A | CYS163, HIS303, HIS340, PHE400, |  |
| 1kfu | L | GLN99, CYS105, HIS262, ASN286, |  |
| 1kra | C | HIS219, ASP221, HIS320, ARG336, |  |
| 1l9f | A | HIS45, ARG49, HIS269, CYS315, |  |
| 1lba | A | LYS128 |  |
| 1lcb | A | GLU60, CYS198, SER219, ASP221, ASP257, HIS259, |  |
| 1lxa | A | HIS125 |  |
| 1mas | A | ASN168, HIS241, |  |
| 1mbb | A | ARG159, SER229, GLU325 |  |
| 1mhl | AC | AGLN91, AHIS95, CARG239 |  |
| 1mhy | D | CYS151, THR213, |  |
| 1mka | AB | AHIS70, AVAL76, AGLY79, ACYS80, BASP84 |  |
| 1mla | A | SER92, HIS201, GLN250 |  |
| 1moq | A | GLU481, LYS485, GLU488, HIS504, LYS603 |  |
| 1mpp | A | ASP32, SER35, TYR75, ASP215, | Replaced by 3pepA |
| 1mpy | A | HIS199, HIS246, TYR255 |  |
| 1nba | A | ASP51, LYS144, ALA172, THR173, CYS177 |  |
| 1nid | A | ASP98, HIS255, |  |
| 1nsp | A | LYS16, ASN119, HIP122 |  |
| 1nzy | A | PHE64, HIS90, GLY114, TRP137, ASP145 |  |
| 1ofg | A | LYS129, TYR217, |  |
| 1pfk | A | GLY11, ARG72, THR125, ASP127, ARG171 |  |
| 1pgs | A | ASP60, GLU206, | Replaced by 1pnfA |
| 1pjb | A | LYS74, HIS95, GLU117, ASP269, |  |
| 1pkn | A | ARG72, ARG119, LYS269, THR327, SER361, GLU363, | Replaced by 1aqfA |
| 1ps1 | A | PHE77, HIS309, |  |
| 1pud | A | ASP102 |  |
| 1pya | AEF | ATYR62, ESER81, FPHE195, FGLU197, |  |
| 1pym | A | GLY47, LEU48, ASP58, LYS120, |  |
| 1qfe | A | GLU86, HIS143, LYS170 |  |
| 1qpr | AB | BARG105, ALYS140, AGLU201, AASP222, |  |
| 1qq5 | A | ASP8, THR12, ARG39, ASN115, LYS147, SER171, ASN173, PHE175, ASP176 |  |
| 1qum | A | GLU261 |  |
| 1r51 | A | ARG176, GLN228, | Replaced by 1uoxA |
| 1ra2 | A | ILE5, MET20, ASP27, LEU28, PHE31, LEU54, ILE94 |  |
| 1rbl | A | LYS175, LYS177, LYS201, ASP203, HIS294, HIS327, |  |
| 1req | A | TYR89, HIS244, LYS604, ASP608, HIS610 |  |
| 1rpt | A | ARG11, HIS12, ARG15, ARG79, HIS257, ASP258, |  |
| 1smn | A | ARG87, HIS89, ASN119 | Replaced by 1qaeA |
| 1tyf | A | GLY68, SER97, MET98, HIS122, ASP171 |  |
| 1uae | A | ASN23, CYS115, ASP305, ARG397, |  |
| 1uag | A | LYS115, ASN138, HIS183 |  |
| 1ula | A | HIS86, GLU89, ASN243 |  |
| 1uok | A | ASP199, GLU255, ASP329 |  |
| 1vao | A | TYR108, ASP170, HIS422, TYR503, ARG504 |  |
| 1vnc | A | LYS353, HIS404, |  |
| 1wgi | A | ASP117 | Replaced by 1hukA |
| 1xva | A | GLU15 |  |
| 1ytw | A | GLU290, ASP356, HIS402, CYS403, ARG409, THR410, |  |
| 1zio | A | LYS13, ARG127, ARG160, ASP162, ASP163, ARG171, |  |
| 2acy | A | ARG23, ASN41, |  |
| 2adm | A | ASN105, PRO106, TYR108 |  |
| 2alr | A | TYR49, LYS79, |  |
| 2bbk | L | ASP32, TRQ57, ASP76, TRP108, TYR119, THR122, |  |
| 2bmi | A | ASP86, ASN176, | Replaced by 1kr3A |
| 2cpo | A | HIS105, GLU183, |  |
| 2hdh | A | SER137, HIS158, GLU170, ASN208, |  |
| 2hgs | A | ARG125, SER151, GLY369, ARG450, |  |
| 2jcw | A | HIS63, ARG143, |  |
| 2pda | A | GLU64 |  |
| 2pfl | A | TRP333, CYS418, CYS419, GLY734, |  |
| 2phk | A | ASP149, LYS151, |  |
| 2plc | A | HIS45, ASP46, ARG84, HIS93, ASP278 |  |
| 2thi | A | CYS113, GLU241, |  |
| 3csm | A | ARG16, ARG157, LYS168, GLU246, |  |
| 3eca | A | THR12, TYR25, THR89, ASP90, LYS162 |  |
| 3pca | M | TYR447, ARG457, |  |
| 4kbp | A | HIS202, HIS295, HIS296 |  |
| 5cox | A | GLN203, HIS207, TYR385 |  |
| 5enl | A | GLU168, GLU211, LYS345, HIS373, |  |
| 5fit | A | GLN83, HIS94, HIS96 |  |
| 8tln | E | GLU143, HIS231, |  |
| 9pap | A | GLN19, OCS25, HIS159, ASN175 |  |
