# Supplementary material for: Accurate Prediction of Protein Catalytic Residues by Side Chain Orientation and Residue Contact Density
Source: PLoS One. 2012 Oct 24;7(10):e47951. doi: 10.1371/journal.pone.0047951 (PMC3480458; doi:10.1371/journal.pone.0047951)
Supplement: Dataset S3 — List of PDB for the EF fold dataset. (DOCX) [file pone.0047951.s006.docx]

Dataset S3: EF fold

| **PDB** | **Chain** | **CSA Annotated Active Site Residues** | **Note** |
| --- | --- | --- | --- |
| 1a26 | A | TYR907, GLU988, |  |
| 1a41 | A | TYR274, ARG223, HIS265, LYS167, |  |
| 1a4i | A | LYS56 |  |
| 1a4y | B | HIS13, LYS40, HIS114 |  |
| 1a6d | A | THR97, ASP390, THR96, ASP63, |  |
| 1a79 | A | LYS156, TYR115, HIS125 |  |
| 1aa6 | A | LYS44, CYS140, HIS141, ARG333, |  |
| 1ab4 | A | TYR122, ARG32, HIS78 |  |
| 1adn | A | CYS69 |  |
| 1af7 | A | ARG98, ASP154, |  |
| 1afr | A | THR199, ASP228, TRP62, HIS146, |  |
| 1afw | A | HIS375, CYS403, CYS125, GLY405, |  |
| 1ak0 | A | ARG48 |  |
| 1ako | A | HIS259, ASP229, ASN7, ASP151, ASN153 |  |
| 1am2 | A | HIS75, ASN198, ASN74, SER1, HIS197, THR72, |  |
| 1aop | A | LYS215, ARG83, CYS483, LYS217, ARG153 |  |
| 1aui | A | ASP121, HIS151, |  |
| 1auk | A | LYS302, ASP281, HIS229, LYS123, GLY69, HIS125, SER150 |  |
| 1b5q | A | GLU62, |  |
| 1b65 | A | ASN218, GLY289, TYR146, SER288, SER250 |  |
| 1b66 | AB | CYS42, GLU133, ASP88, HIS89, | Replaced by 1gtq |
| 1b93 | A | HIS98, ASP71, HIS19, ASP101, ASP91, GLY66, |  |
| 1bd3 | A | THR141, ARG137, ASP235 |  |
| 1bfd | A | GLU28, HIS281, HIS70 |  |
| 1bg6 | A | ASP297, HIS202, |  |
| 1bmt | A | SER810, ASP757, HIS759 |  |
| 1bo1 | A | ASP278, LYS150, |  |
| 1bob | A | GLU255 |  |
| 1bol | A | HIS109, HIS46, GLU105 |  |
| 1bou | B | HIS195 |  |
| 1brw | A | LYS187, SER183, HIS82, ARG168, |  |
| 1bwd | A | ASP179, ASP229, HIS331, ARG127, CYS332, ASP108, HIS227 |  |
| 1c17 | A | ASP61, SER206, ASN214, ARG210, |  |
| 1c3c | AB | THR140, HIS141, LYS268, GLU275, | Replaced by 2x75 |
| 1c4x | A | HIS263, SER110, ASP235 |  |
| 1c4z | A | GLU550, ARG506, CYS820, HIS818, GLU539, ASP607, |  |
| 1cb8 | A | TYR234, HIS225, ARG288 |  |
| 1cc1 | LS | THR19, CYS492, GLU23 |  |
| 1cf2 | O | CYS140, HIS219, |  |
| 1cfr | A | LYS190 |  |
| 1cg2 | A | GLU175, GLU200, HIS112, ASP141, HIS385, GLU176, |  |
| 1chd | A | ASP286, SER164, MET283, THR165, HIS190 |  |
| 1chk | B | GLU22, ASP40, |  |
| 1chm | A | GLU262, GLU358, HIS232 |  |
| 1cjy | A | SER228, GLY198, GLY197, ASP549, |  |
| 1cqq | A | GLY145, HIS40, GLU71, CYS147, |  |
| 1cs1 | AC | LYS198, TYR101, ASP173, ARG48, |  |
| 1d1q | A | ALA13, ARG19, SER20, ASP132, |  |
| 1d4a | A | TYR155, HIS161, GLY149 |  |
| 1d5r | A | ASP92, ARG130, CYS124 |  |
| 1dci | A | ASP204, GLU196, |  |
| 1dgs | A | ASP118, LYS116, LYS312, ARG196, |  |
| 1di1 | A | TYR92, PHE112, TRP333, PHE178, |  |
| 1dj0 | A | ASP60 |  |
| 1dki | A | SER47, HIS195, |  |
| 1dl2 | A | ARG136, GLU435, ASP275, GLU132, | Use atom CB as side chain vector atom for residue R75, K409 and K539 |
| 1dl5 | A | SER59 |  |
| 1dnp | A | TRP306, TRP359, TRP382 |  |
| 1dqa | AB | LYS691, ASP767, GLU559, HIS866, |  |
| 1dqs | A | HIS275 |  |
| 1dw9 | AF | GLU99, ARG96, |  |
| 1e0c | A | GLN231, HIS234, HIS233, ARG235, THR232, CYS230, |  |
| 1e19 | A | LYS215, LYS277, LYS131 |  |
| 1e7l | AB | HIS43, GLU65, HIS41, HIS105 |  |
| 1ecf | A | GLY102, CYS1, ASN101 |  |
| 1ecm | AB | ARG28, ARG11, |  |
| 1eej | A | CYS98, TYR100, CYS101, ARG125, |  |
| 1eg7 | A | LYS1074 |  |
| 1ehk | AB | TYR237, HIS386, HIS233, HIS384, ARG449, PHE385, ARG450, PHE86, PHE88 |  |
| 1ei5 | A | ASN155, HIS287, SER62, LYS65, TYR153 | Replaced by 1cefA |
| 1et0 | A | THR38, GLU193, PLP413, LYS159, |  |
| 1eu1 | A | TYR114, TRP116, |  |
| 1eul | A | ASP351 |  |
| 1f6d | A | GLU131, ASP95, GLU117, HIS213, |  |
| 1f75 | A | ARG42, ARG33, ARG197, ARG203, |  |
| 1f7l | A | LYS62, HIS105 |  |
| 1f7u | A | LYS156, HIS162, HIS159 |  |
| 1fft | F | HIS419, LYS362, ASP135, THR211, THR359, SER315, SER299, ASN142, TYR61, THR201, SER145, MET79, ASN124, PHE420, PHE103, GLU286, THR149, HIS284, HIS421, THR204, ASP75, ARG71, TYR288 |  |
| 1fgj | AB | TYR334, HIS268, ASP267, TYR467, |  |
| 1foh | A | ASP54, ARG281, TYR289 |  |
| 1fr2 | B | HIS102, HIS103, GLU100, ARG5, |  |
| 1fui | A | GLU337, ASP361, |  |
| 1g0d | A | CYS272, ASP355, HIS332, TYR515, |  |
| 1g24 | A | GLU214 |  |
| 1g72 | A | ASP297 |  |
| 1g8f | A | ARG290 |  |
| 1ga8 | A | GLN189, ASP130, ASN153 |  |
| 1gpj | A | SER48, HIS84, |  |
| 1gpm | A | HIS181, GLU183, TYR87, CYS86, GLY59, ASP239, |  |
| 1gpr | A | GLY85, HIS68, HIS83, THR66, |  |
| 1gq8 | A | ASP136, GLN113, GLN135, ASP157, |  |
| 1gsa | A | ARG225, LYS160, ARG210 |  |
| 1gt7 | A | GLU117, GLU171 |  |
| 1h3i | A | TYR335, HIS293, |  |
| 1h4g | A | GLU184, GLU94, |  |
| 1h54 | A | GLU487 |  |
| 1hfe | M | LYS237, CYS178, |  |
| 1hr6 | B | GLU73, |  |
| 1hrk | A | GLU343, HIS263, HIS341 |  |
| 1hv9 | A | ARG18 | Replaced by 2w0wA, remove terminal incomplete residue K454 |
| 1i19 | A | GLU311, GLU475, ARG477, |  |
| 1i6p | A | ASP44, ARG46, |  |
| 1i78 | A | HIS212, ASP85, ASP83, ALA99, ASP210 |  |
| 1i8d | A | HIS102, CYS48, MET64, PHE2, SER41 |  |
| 1i8t | B | ASP348, ARG278, GLU298, ARG170, |  |
| 1inp | A | LYS37, THR158, |  |
| 1itx | A | GLU204, ASP200, |  |
| 1j09 | A | LYS246 |  |
| 1jch | A | ASP510, HIS513, ARG545, GLU517, |  |
| 1jfl | A | CYS82, CYS194, |  |
| 1jh6 | A | HIS42, SER121, MET117, HIS119, TYR124 |  |
| 1jhf | A | MET118, GLU152, LYS156, SER119, ASP127 |  |
| 1jm6 | A | HIS1239, GLU1243, |  |
| 1jms | A | ASP434 |  |
| 1jnr | AB | ASP361, TRP234, SER449, ASN74, GLU141, ARG265, HIS398, TRP48 |  |
| 1k30 | A | HIS139, ASP144, |  |
| 1k32 | A | SER965, HIS746, ASP966, GLY918, |  |
| 1k4t | A | HIS632, ARG488, ARG590, TYR723, |  |
| 1k82 | A | GLU2, LYS56, PRO1, ARG258, |  |
| 1kl7 | A | PLP1400, LYS124, |  |
| 1kny | A | GLU145, LYS149, |  |
| 1kyq | A | ASP141 |  |
| 1kzh | A | THR204, ASP206, GLY82, LYS203, ARG146 |  |
| 1l0o | A | GLU46, ARG105 |  |
| 1l1d | A | HIS480, ARG493, CYS495, CYS440, ASP484 |  |
| 1l1l | A | CYS408, CYS419, ASN406, GLU410, CYS119 | Replaced by 1xjeA |
| 1lam | A | LYS262, ARG336, ASP255 |  |
| 1lba | A | TYR46, LYS128, |  |
| 1lbu | A | HIS192 |  |
| 1lci | A | HIS245, ARG218, LYS529, THR343, |  |
| 1ldm | A | HIS193, ASP166, ARG169 |  |
| 1m6k | A | SER67, LYS70, |  |
| 1mka | AB | CYS80, VAL76, GLY79, HIS70, ASP84 |  |
| 1moq | A | LYS603, LYS485, GLU488, GLU481, |  |
| 1mpx | A | TYR175, HIS340, SER174, TYR82, ASP307 |  |
| 1mpy | A | HIS199, HIS246, TYR255 |  |
| 1mt5 | A | LYS142, SER218, ILE238, SER241, GLY240, GLY239, SER217 | Replaced by 2vyaA |
| 1muc | A | LYS169, LYS167, GLU327 |  |
| 1mug | A | ASN18 |  |
| 1nf9 | A | ASP38 |  |
| 1ni4 | AD | HIS263, GLU59, HIS128 |  |
| 1nvm | A | TYR291, HIS21, |  |
| 1nww | A | TYR53, ARG99, ASP101, ASN55, ASP132 |  |
| 1o04 | A | SER302, LYS192, GLU268, GLU399, |  |
| 1o8a | A | GLU384, HIS353, HIS513, ALA354, TYR523, |  |
| 1o98 | A | SER62, ASP154, ARG261 |  |
| 1oac | A | ASP383, TYR466, | Replaced by 1dyuA |
| 1oe8 | A | TYR10 |  |
| 1otg | A | PRO2, PHE35, ARG41 |  |
| 1p3d | A | LYS129 |  |
| 1pgs | A | ASP60, GLU206, | Replaced by 1pnfA |
| 1pmi | A | GLN111, ARG304, GLU294 |  |
| 1pvd | A | ASP28, HIS114, HIS115, GLU477, |  |
| 1pya | AEF | TYR62, PHE195, GLU197, SER81, |  |
| 1pym | A | ASP58, LEU48, GLY47, LYS120, |  |
| 1qba | A | GLU540, ASP539, |  |
| 1qdl | AB | HIS306, HIS175, GLU177, CYS84, |  |
| 1qf6 | A | ARG363 |  |
| 1qfm | A | ASP641, SER554, HIS680 |  |
| 1qh5 | A | ASP58, |  |
| 1qho | A | ASP228, ASP329, GLU256, HIS132, |  |
| 1qi9 | A | HIS411, HIS418, HIS486, LYS341 |  |
| 1qmh | A | HIS309 |  |
| 1qq5 | A | ARG39, THR12, ASP176, PHE175, ASP8, ASN173, LYS147, ASN115, SER171 |  |
| 1qtn | A | GLY350, HIS317, CYS360, ARG258, |  |
| 1rtu | A | GLU62, HIS101, HIS41 |  |
| 1sox | A | ARG138, TYR322, | no proper homologous protein, use atom CB for D85, E86, Q94 |
| 1tml | A | ASP117, ASP265, |  |
| 1uaq | A | GLU64, CYS91, SER89, |  |
| 1ush | A | ARG375, ASP120, ARG410, HIS117, ARG379, ASN116, |  |
| 1xva | A | GLU15 |  |
| 1zym | A | HIS189, THR168, |  |
| 2abk | A | ASP138, LYS120, |  |
| 2ahj | AB | ARG56, SER113, CYS114, CYS112, | Replaced by 1ahj |
| 2apr | A | THR221, SER38, ASP35, ASP218, |  |
| 2bbk | L | ASP76, THR122, ASP32, TYR119, TRP57, TRP108, |  |
| 2pgd | A | ASN187, LYS183, GLY130, GLU190, |  |
| 2pia | A | CYS199, ASN44, SER58, GLU223, |  |
| 3cla | A | ASP199, HIS195, ARG18 | Replaced by 1q23A |
| 3mdd | A | GLU376 |  |
| 7odc | A | GLU274, LYS69, HIS197 |  |
