# Supplementary material for: Accurate Prediction of Protein Catalytic Residues by Side Chain Orientation and Residue Contact Density
Source: PLoS One. 2012 Oct 24;7(10):e47951. doi: 10.1371/journal.pone.0047951 (PMC3480458; doi:10.1371/journal.pone.0047951)
Supplement: Dataset S5 — List of PDB for the EF family dataset. (DOCX) [file pone.0047951.s008.docx]

Dataset S5: EF family

| **PDB** | **Chain** | **CSA Annotated Active Site Residues** | **Note** |
| --- | --- | --- | --- |
| 12as | A | ARG100, ASP46, GLN116 |  |
| 1a0i | A | LYS34 |  |
| 1a26 | A | TYR907, GLU988, |  |
| 1a4i | A | LYS56 |  |
| 1a4y | B | HIS13, LYS40, HIS114 |  |
| 1a6d | A | THR97, ASP390, THR96, ASP63, |  |
| 1a79 | A | LYS156, TYR115, HIS125 |  |
| 1a8h | A | LYS300, LYS297, |  |
| 1aa6 | A | LYS44, CYS140, HIS141, ARG333, |  |
| 1ab4 | A | TYR122, ARG32, HIS78 |  |
| 1adn | A | CYS69 |  |
| 1af7 | A | ARG98, ASP154, |  |
| 1afr | A | THR199, ASP228, TRP62, HIS146, |  |
| 1afw | A | HIS375, CYS403, CYS125, GLY405, |  |
| 1ah7 | A | ASP55 |  |
| 1ak0 | A | ARG48 |  |
| 1ako | A | HIS259, ASP229, ASN7, ASP151, ASN153 |  |
| 1am2 | A | HIS75, ASN198, ASN74, SER1, HIS197, THR72, |  |
| 1aop | A | LYS215, ARG83, CYS483, LYS217, ARG153 |  |
| 1apy | AB | SER49, THR201, THR234, THR183, GLY235 |  |
| 1ast | A | TYR149, GLU93, |  |
| 1aug | A | GLU81, CYS144, ARG91, HIS168, |  |
| 1aui | A | ASP121, HIS151, |  |
| 1auo | A | HIS199, SER114, GLN115, LEU23, ASP168 |  |
| 1avq | A | LYS131 |  |
| 1aw8 | B | LYS9, TYR58, PYR25 |  |
| 1ax4 | A | LYS266, ASP223, PHE132 |  |
| 1b04 | A | ALA114 |  |
| 1b5q | A | GLU62, |  |
| 1b5t | A | GLU28, ASP120, |  |
| 1b65 | A | ASN218, GLY289, TYR146, SER288, SER250 |  |
| 1b66 | AB | CYS42, GLU133, ASP88, HIS89, | Replaced by 1gtq |
| 1b6g | A | HIS289, ASP124, ASP260 |  |
| 1b93 | A | HIS98, ASP71, HIS19, ASP101, ASP91, GLY66, |  |
| 1bd0 | AB | ARG136, LYS39, CYS311, TYR265, |  |
| 1bf2 | A | ASP510, ASP375, GLU435 |  |
| 1bfd | A | GLU28, HIS281, HIS70 |  |
| 1bg6 | A | ASP297, HIS202, |  |
| 1bhg | A | TYR504, GLU540, GLU451 |  |
| 1bmt | A | SER810, ASP757, HIS759 |  |
| 1bo1 | A | ASP278, LYS150, |  |
| 1bob | A | GLU255 |  |
| 1bol | A | HIS109, HIS46, GLU105 |  |
| 1boo | A | PRO54, SER53, ASP96 |  |
| 1bou | B | HIS195 |  |
| 1brw | A | LYS187, SER183, HIS82, ARG168, |  |
| 1bzy | A | GLU133, ASP134, ARG169, ASP137, LYS165 |  |
| 1c17 | A | ASP61, SER206, ASN214, ARG210, |  |
| 1c3c | AB | THR140, HIS141, LYS268, GLU275, | Replaced by 2x75 |
| 1c4x | A | HIS263, SER110, ASP235 |  |
| 1c4z | A | GLU550, ARG506, CYS820, HIS818, GLU539, ASP607, |  |
| 1cb8 | A | TYR234, HIS225, ARG288 |  |
| 1cc1 | LS | THR19, CYS492, GLU23 |  |
| 1cfr | A | LYS190 |  |
| 1cg2 | A | GLU175, GLU200, HIS112, ASP141, HIS385, GLU176, |  |
| 1chd | A | ASP286, SER164, MET283, THR165, HIS190 |  |
| 1chk | B | GLU22, ASP40, |  |
| 1chm | A | GLU262, GLU358, HIS232 |  |
| 1cjy | A | SER228, GLY198, GLY197, ASP549, |  |
| 1cmx | A | ASP181, CYS90, HIS166, GLN84, |  |
| 1cqq | A | GLY145, HIS40, GLU71, CYS147, |  |
| 1cvr | A | GLY212, GLU152, HIS211, CYS244, |  |
| 1d1q | A | ALA13, ARG19, SER20, ASP132, |  |
| 1d2t | A | HIS189, ASP193, ARG183, HIS150, |  |
| 1d4a | A | TYR155, HIS161, GLY149 |  |
| 1d5r | A | ASP92, ARG130, CYS124 |  |
| 1d8c | A | ARG338, GLU272, ASP270, ASP631, | Replaced by 2jqxA |
| 1dbt | A | ASP60, LYS62, |  |
| 1dci | A | ASP204, GLU196, |  |
| 1dek | A | ARG68, HIS206, |  |
| 1dgs | A | ASP118, LYS116, LYS312, ARG196, |  |
| 1di1 | A | TYR92, PHE112, TRP333, PHE178, |  |
| 1din | A | CYS123, ASP171, HIS202 |  |
| 1dj0 | A | ASP60 |  |
| 1dki | A | SER47, HIS195, |  |
| 1dl2 | A | ARG136, GLU435, ASP275, GLU132, | Use atom CB as side chain vector atom for residue R75, K409 and K539 |
| 1dl5 | A | SER59 |  |
| 1dmu | A | LYS144 |  |
| 1dnp | A | TRP306, TRP359, TRP382 |  |
| 1dpg | A | ASP177, HIS240, |  |
| 1dqa | AB | LYS691, ASP767, GLU559, HIS866, |  |
| 1dqs | A | HIS275 |  |
| 1dtw | AB | HIS291, GLU76, |  |
| 1dw9 | AF | GLU99, ARG96, |  |
| 1dxe | A | ARG75, HIS50, |  |
| 1e19 | A | LYS215, LYS277, LYS131 |  |
| 1e1a | A | HIS287, GLU37 |  |
| 1e2t | A | HIS107, ASP122, CYS69 |  |
| 1e5q | A | ASP126, |  |
| 1e7l | AB | HIS43, GLU65, HIS41, HIS105 |  |
| 1eb6 | A | TYR106, GLU129, |  |
| 1ecm | AB | ARG28, ARG11, |  |
| 1eej | A | CYS98, TYR100, CYS101, ARG125, |  |
| 1eg7 | A | LYS1074 |  |
| 1ehk | AB | TYR237, HIS386, HIS233, HIS384, ARG449, PHE385, ARG450, PHE86, PHE88 |  |
| 1ehy | A | TRP38, TYR215, TYR152, ASP107, ASP246, HIS275, PHE108 |  |
| 1ei5 | A | ASN155, HIS287, SER62, LYS65, TYR153 | Replaced by 1cefA |
| 1eq2 | A | TYR140, LYS144, SER116 |  |
| 1esc | A | TRP280, SER14, HIS283 |  |
| 1et0 | A | THR38, GLU193, PLP413, LYS159, |  |
| 1eu1 | A | TYR114, TRP116, |  |
| 1eul | A | ASP351 |  |
| 1f2d | A | THR81, LYS51, GLU296, SER78, TYR295 |  |
| 1f2v | A | HIS43 |  |
| 1f6d | A | GLU131, ASP95, GLU117, HIS213, |  |
| 1f75 | A | ARG42, ARG33, ARG197, ARG203, |  |
| 1f7l | A | LYS62, HIS105 |  |
| 1f7u | A | LYS156, HIS162, HIS159 |  |
| 1f8m | A | ARG228, HIS180, CYS191 |  |
| 1f8r | A | LYS326, HIS223, |  |
| 1fa0 | A | LYS215, |  |
| 1fcq | A | ASP111, GLU113, TYR184, TYR227, TRP301 |  |
| 1fft | F | HIS419, LYS362, ASP135, THR211, THR359, SER315, SER299, ASN142, TYR61, THR201, SER145, MET79, ASN124, PHE420, PHE103, GLU286, THR149, HIS284, HIS421, THR204, ASP75, ARG71, TYR288 |  |
| 1fgj | AB | TYR334, HIS268, ASP267, TYR467, |  |
| 1foh | A | ASP54, ARG281, TYR289 |  |
| 1fr2 | B | HIS102, HIS103, GLU100, ARG5, |  |
| 1fui | A | GLU337, ASP361, |  |
| 1g0d | A | CYS272, ASP355, HIS332, TYR515, |  |
| 1g24 | A | GLU214 |  |
| 1g72 | A | ASP297 |  |
| 1g8f | A | ARG290 |  |
| 1g8p | A | ARG289 |  |
| 1g99 | A | GLY212, SER10, ASP148, ARG91, ARG241 |  |
| 1ga8 | A | GLN189, ASP130, ASN153 |  |
| 1gdo | B | ASN98, GLY99, CYS1 |  |
| 1gpj | A | SER48, HIS84, |  |
| 1gpm | A | HIS181, GLU183, TYR87, CYS86, GLY59, ASP239, |  |
| 1gpr | A | GLY85, HIS68, HIS83, THR66, |  |
| 1gq8 | A | ASP136, GLN113, GLN135, ASP157, |  |
| 1gsa | A | ARG225, LYS160, ARG210 |  |
| 1gt7 | A | GLU117, GLU171 |  |
| 1h3i | A | TYR335, HIS293, |  |
| 1h4g | A | GLU184, GLU94, |  |
| 1h54 | A | GLU487 |  |
| 1hdh | A | ALA51, LYS375, HIS211, LYS113, ASP317, ARG55, HIS115, | Replaced by 1fsuA |
| 1hfe | M | LYS237, CYS178, |  |
| 1hr6 | B | GLU73, |  |
| 1hrk | A | GLU343, HIS263, HIS341 |  |
| 1hv9 | A | ARG18 | Replaced by 2w0wA, remove terminal incomplete residue K454 |
| 1hzf | A | CYS991, GLN994, |  |
| 1i19 | A | GLU311, GLU475, ARG477, |  |
| 1i1i | P | TYR613, GLU503 |  |
| 1i6p | A | ASP44, ARG46, |  |
| 1i78 | A | HIS212, ASP85, ASP83, ALA99, ASP210 |  |
| 1i7q | AB | HIS398, HIS172, CYS85, GLU174, |  |
| 1i8d | A | HIS102, CYS48, MET64, PHE2, SER41 |  |
| 1i8t | B | ASP348, ARG278, GLU298, ARG170, |  |
| 1ig8 | A | ASP211, SER158, ARG173 |  |
| 1im5 | A | ASP10, CYS133, ALA129, LYS94 |  |
| 1inp | A | LYS37, THR158, |  |
| 1itx | A | GLU204, ASP200, |  |
| 1iu4 | A | TRP272, CYS64, LYS269, HIS274, ASP255 |  |
| 1ivh | A | GLU254 |  |
| 1j09 | A | LYS246 |  |
| 1j79 | A | ASP250 |  |
| 1jch | A | ASP510, HIS513, ARG545, GLU517, |  |
| 1jdw | A | ASP254, CYS407, HIS303 |  |
| 1jfl | A | CYS82, CYS194, |  |
| 1jh6 | A | HIS42, SER121, MET117, HIS119, TYR124 |  |
| 1jhf | A | MET118, GLU152, LYS156, SER119, ASP127 |  |
| 1jkm | A | HIS338, ASP308, SER202 |  |
| 1jm6 | A | HIS1239, GLU1243, |  |
| 1jms | A | ASP434 |  |
| 1jnr | AB | ASP361, TRP234, SER449, ASN74, GLU141, ARG265, HIS398, TRP48 |  |
| 1jof | A | GLU212, HIS148, ARG274, ARG196, | no existing homologus protein, use atom CB for K29, E38, K68, Q128, R173, R246, Q249, K252 |
| 1jqn | A | HIS138, ARG713, ARG699, ARG581, ARG396 |  |
| 1k30 | A | HIS139, ASP144, |  |
| 1k32 | A | SER965, HIS746, ASP966, GLY918, |  |
| 1k4t | A | HIS632, ARG488, ARG590, TYR723, |  |
| 1k82 | A | GLU2, LYS56, PRO1, ARG258, |  |
| 1kdg | A | HIS689, ASN732, |  |
| 1kny | A | GLU145, LYS149, |  |
| 1kws | A | GLU281, |  |
| 1kyq | A | ASP141 |  |
| 1kzh | A | THR204, ASP206, GLY82, LYS203, ARG146 |  |
| 1l0o | A | MG602, GLU46, ARG105 |  |
| 1l1d | A | HIS480, ARG493, CYS495, CYS440, ASP484 |  |
| 1l1l | A | CYS408, CYS419, ASN406, GLU410, CYS119 | Replaced by 1xjeA |
| 1l6p | A | CYS109, CYS103, TYR42, ASP68, TYR71, PHE70, |  |
| 1l7d | A | ASP135, SER138, ARG127, GLN132, | Replaced by 1f8gB |
| 1lam | A | LYS262, ARG336, ASP255 |  |
| 1lba | A | TYR46, LYS128, |  |
| 1lbu | A | HIS192 |  |
| 1lci | A | HIS245, ARG218, LYS529, THR343, |  |
| 1ldm | A | HIS193, ASP166, ARG169 |  |
| 1lml | A | GLU265 |  |
| 1ltq | A | LYS15, ARG126, ASP35, ASP165, |  |
| 1luc | A | HIS44, HIS45, PHE261 |  |
| 1lxa | A | HIS125 |  |
| 1m53 | A | ASP241, GLU295, |  |
| 1m6k | A | SER67, LYS70, |  |
| 1mek | A | HIS38, CYS39, GLY37, CYS36, |  |
| 1mka | AB | CYS80, VAL76, GLY79, HIS70, ASP84 |  |
| 1mla | A | HIS201, SER92, GLN250 |  |
| 1moq | A | LYS603, LYS485, GLU488, GLU481, |  |
| 1mpp | A | ASP215, TYR75, SER35, ASP32, | Replaced by 3pepA |
| 1mpx | A | TYR175, HIS340, SER174, TYR82, ASP307 |  |
| 1mpy | A | HIS199, HIS246, TYR255 |  |
| 1mt5 | A | LYS142, SER218, ILE238, SER241, GLY240, GLY239, SER217 | Replaced by 2vyaA |
| 1muc | A | LYS169, LYS167, GLU327 |  |
| 1mug | A | ASN18 |  |
| 1nba | A | ALA172, ASP51, CYS177, THR173, LYS144 |  |
| 1nbf | A | ASN218, CYS223, ASP481, HIS464, |  |
| 1ni4 | AD | HIS263, GLU59, HIS128 |  |
| 1nvm | A | TYR291, HIS21, |  |
| 1nww | A | TYR53, ARG99, ASP101, ASN55, ASP132 |  |
| 1o04 | A | SER302, LYS192, GLU268, GLU399, |  |
| 1o98 | A | SER62, ASP154, ARG261 |  |
| 1oac | A | ASP383, TYR466, | Replaced by 1dyuA |
| 1oe8 | A | TYR10 |  |
| 1ofd | A | LYS972, GLU903, CYS1 |  |
| 1otg | A | PRO2, PHE35, ARG41 |  |
| 1p3d | A | LYS129 |  |
| 1pbg | A | GLU375, GLU160, |  |
| 1pgs | A | ASP60, GLU206, | Replaced by 1pnfA |
| 1pii | A | SER215, LYS114, GLU53, ASN184, LYS55, GLU163, |  |
| 1pma | B | GLY47, LYS33, THR1, SER129, |  |
| 1pmi | A | GLN111, ARG304, GLU294 |  |
| 1psd | A | GLU269, HIS292, |  |
| 1pvd | A | ASP28, HIS114, HIS115, GLU477, |  |
| 1pya | AEF | TYR62, PHE195, GLU197, SER81, |  |
| 1qam | A | GLY38, ASN101, GLU59 |  |
| 1qaz | A | TYR246, ASN191, ARG239 |  |
| 1qba | A | GLU540, ASP539, |  |
| 1qd1 | A | HIS82 |  |
| 1qd6 | CD | GLY146, HIS142, SER144 |  |
| 1qdl | AB | HIS306, HIS175, GLU177, CYS84, |  |
| 1qe3 | A | GLU310, SER189, HIS399 |  |
| 1qf6 | A | ARG363 |  |
| 1qfe | A | LYS170, HIS143, GLU86 |  |
| 1qfm | A | ASP641, SER554, HIS680 |  |
| 1qfn | A | TYR13, ARG8, GLY10, LYS18, TYR72 |  |
| 1qgn | A | ASP236, LYS261, TYR163 | Replaced by 1i43A |
| 1qh5 | A | ASP58, |  |
| 1qho | A | ASP228, ASP329, GLU256, HIS132, |  |
| 1qi9 | A | HIS411, VO4600, HIS418, HIS486, LYS341 |  |
| 1qj4 | A | SER80, ASP207, HIS235 |  |
| 1qmh | A | HIS309 |  |
| 1qq5 | A | ARG39, THR12, ASP176, PHE175, ASP8, ASN173, LYS147, ASN115, SER171 |  |
| 1qtn | A | GLY350, HIS317, CYS360, ARG258, |  |
| 1rhs | A | ARG248, ARG186, LYS249, VAL251, CYS247, GLY250, THR252 |  |
| 1rtu | A | GLU62, HIS101, HIS41 |  |
| 1slm | A | GLU202 |  |
| 1sml | A | TYR191 |  |
| 1sox | A | ARG138, TYR322, | no proper homologous protein, use atom CB for D85, E86, Q94 |
| 1tdj | A | SER315, LYS62, |  |
| 1tht | A | SER114, HIS241, |  |
| 1tml | A | ASP117, ASP265, |  |
| 1tyf | A | MET98, SER97, GLY68, HIS122, ASP171 |  |
| 1uaq | A | GLU64, CYS91, SER89, |  |
| 1uro | A | TYR164, ASP86, |  |
| 1ush | A | ARG375, ASP120, ARG410, HIS117, ARG379, ASN116, |  |
| 1vie | A | ILE68, LYS32, TYR69, GLN67, |  |
| 1xva | A | GLU15 |  |
| 1zym | A | HIS189, THR168, |  |
| 2abk | A | ASP138, LYS120, |  |
| 2acy | A | ARG23, ASN41, |  |
| 2ahj | AB | ARG56, SER113, CYS114, CYS112, | Replaced by 1ahj |
| 2ayh | A | GLU109, ASP107, GLU105 |  |
| 2bbk | L | ASP76, THR122, ASP32, TYR119, TRP57, TRP108, |  |
| 2ebn | A | ASP130, GLU132, |  |
| 2eng | A | ASP121, ASP10, | Replaced by 1l8fA |
| 2fok | A | LYS469 |  |
| 2gsa | A | LYS273, ASP245, TYR150 |  |
| 2hgs | A | SER151, GLY369, ARG125, ARG450, |  |
| 2nac | A | GLN313, HIS332, ASN146, ARG284, |  |
| 2pgd | A | ASN187, LYS183, GLY130, GLU190, |  |
| 2pia | A | CYS199, ASN44, SER58, GLU223, |  |
| 2plc | A | ASP278, ARG84, ASP46, HIS45, HIS93 |  |
| 2pth | A | ASN10, ASP93, HIS20 |  |
| 2sqc | A | PHE605, PHE601, GLN262, ASP374, ASP377, GLU45, HIS451, ARG127, TRP312, TYR495, TRP489, TRP169, CYS376, GLU93, PHE365 |  |
| 2tps | A | ARG59, SER130, LYS159 |  |
| 3cla | A | ASP199, HIS195, ARG18 | Replaced by 1q23A |
| 3mdd | A | GLU376 |  |
| 4kbp | A | HIS296, HIS202, HIS295 |  |
| 7odc | A | GLU274, LYS69, HIS197 |  |
