# Supplementary material for: Accurate Prediction of Protein Catalytic Residues by Side Chain Orientation and Residue Contact Density
Source: PLoS One. 2012 Oct 24;7(10):e47951. doi: 10.1371/journal.pone.0047951 (PMC3480458; doi:10.1371/journal.pone.0047951)
Supplement: Dataset S6 — List of PDB for the UB78 dataset. (DOCX) [file pone.0047951.s009.docx]

Dataset S6: UB78

| **PDB** | **Chain** | **CSA Annotated Active Site Residues** | Note |
| --- | --- | --- | --- |
| 1a4s | A | ASN166, GLU263, CYS297 |  |
| 1a4y | B | HIS13, LYS40, HIS114 |  |
| 1a6d | A | THR97, ASP390, THR96, ASP63, |  |
| 1a8q | A | TRP28, MET95, ASP223, SER94, HIS252 |  |
| 1ab4 | A | TYR122, ARG32, HIS78 |  |
| 1akm | A | ARG 106, HIS 133, GLN 136, ASP 231, CYS 273, ARG 319, |  |
| 1ako | A | HIS259, ASP229, ASN7, ASP151, ASN153 |  |
| 1am2 | A | HIS75, ASN198, ASN74, SER1, HIS197, THR72, |  |
| 1aug | A | GLU81, CYS144, ARG91, HIS168, |  |
| 1auo | A | HIS199, SER114, GLN115, LEU23, ASP168 |  |
| 1b04 | A | ALA114 |  |
| 1b65 | A | ASN218, GLY289, TYR146, SER288, SER250 |  |
| 1b6b | A | SER97, LEU111, HIS122, LEU124, TYR168 |  |
| 1b73 | A | ASP7, SER8, CYS70, CYS178, |  |
| 1bg6 | A | ASP297, HIS202, |  |
| 1bml | A | HIS603, ASP646, |  |
| 1bo1 | A | ASP278, LYS150, |  |
| 1bol | A | HIS109, HIS46, GLU105 |  |
| 1brm | A | CYS135, GLN162, HIS274 |  |
| 1bwd | A | ASP179, ASP229, HIS331, ARG127, CYS332, ASP108, HIS227 |  |
| 1bwp | A | SER47, GLY74, ASN104, ASP192, HIS195 |  |
| 1c17 | A | ASP61, SER206, ASN214, ARG210, |  |
| 1c4x | A | HIS263, SER110, ASP235 |  |
| 1c4z | A | GLU550, ARG506, CYS820, HIS818, GLU539, ASP607, |  |
| 1cd5 | A | ASP72, ASP141, HIS143, GLU148, |  |
| 1cfr | A | LYS190 |  |
| 1chd | A | ASP286, SER164, MET283, THR165, HIS190 |  |
| 1chk | A | GLU22, ASP40, |  |
| 1chk | B | GLU22, ASP40, |  |
| 1cz1 | A | GLU192, GLU292, |  |
| 1db3 | A | THR132, GLU134, TYR156, LYS160, |  |
| 1dco | A | HIS62, HIS63, HIS80, ASP89, |  |
| 1di1 | A | TYR92, PHE112, TRP333, PHE178, |  |
| 1e2t | A | HIS107, ASP122, CYS69 |  |
| 1esc | A | TRP280, SER14, HIS283 |  |
| 1eug | A | ASP64, HIS187, |  |
| 1f2v | A | HIS43 |  |
| 1fcq | A | ASP111, GLU113, TYR184, TYR227, TRP301 |  |
| 1fug | AB | BHIS14, BLYS165, BARG244, BLYS245, ALYS265, ALYS269, AASP271 |  |
| 1g24 | A | GLU214 |  |
| 1g8p | A | ARG289 |  |
| 1geq | A | ASP47, TYR161, GLU36 |  |
| 1gpr | A | GLY85, HIS68, HIS83, THR66, |  |
| 1hzd | A | GLU209, GLY186, GLU189 |  |
| 1i8d | A | HIS102, CYS48, MET64, PHE2, SER41 |  |
| 1iu4 | A | TRP272, CYS64, LYS269, HIS274, ASP255 |  |
| 1jfl | A | CYS82, CYS194, |  |
| 1jkm | A | HIS338, ASP308, SER202 |  |
| 1k30 | A | HIS139, ASP144, |  |
| 1k32 | A | SER965, HIS746, ASP966, GLY918, |  |
| 1kas | A | CYS163, HIS303, HIS340, PHE400, |  |
| 1kfu | L | GLN99, CYS105, HIS262, ASN286, |  |
| 1kra | C | HIS219, ASP221, HIS320, ARG336, |  |
| 1l1l | A | CYS408, CYS419, ASN406, GLU410, CYS119 | Use atom CB as side chain vector atom for residue E4, N207, E263, N316, K721 |
| 1l6p | A | CYS109, CYS103, TYR42, ASP68, TYR71, PHE70, |  |
| 1l7d | A | ASP135, SER138, ARG127, GLN132, | Use atom CB as side chain vector atom for residue K245, T351, K352 |
| 1lci | A | HIS245, ARG218, LYS529, THR343, |  |
| 1lxa | A | HIS125 |  |
| 1m53 | A | ASP241, GLU295, |  |
| 1mek | A | HIS38, CYS39, GLY37, CYS36, |  |
| 1mla | A | HIS201, SER92, GLN250 |  |
| 1pgs | A | ASP60, GLU206, | Use atom CB as side chain vector atom for residue D4, N5 |
| 1pjb | A | LYS74, HIS95, GLU117, ASP269, |  |
| 1pma | B | GLY47, LYS33, THR1, SER129, |  |
| 1qdl | AB | AHIS306, BHIS175, BGLU177, BCYS84, |  |
| 1qfn | A | TYR13, ARG8, GLY10, LYS18, TYR72 |  |
| 1smn | A | ARG87, HIS89, ASN119 |  |
| 1tht | A | SER114, HIS241, |  |
| 1tyf | A | MET98, SER97, GLY68, HIS122, ASP171 |  |
| 1uch | A | CYS95, HIS169, ASP184, GLN89, |  |
| 1uok | A | ASP199, GLU255, ASP329 |  |
| 1vie | A | ILE68, LYS32, TYR69, GLN67, |  |
| 1zym | A | HIS189, THR168, |  |
| 2alr | A | TYR49, LYS79, |  |
| 2eng | A | ASP121, ASP10, |  |
| 2fok | A | LYS469 |  |
| 2plc | A | ASP278, ARG84, ASP46, HIS45, HIS93 |  |
| 2pth | A | ASN10, ASP93, HIS20 |  |
